# Estrogenic Plants: to Prevent Neurodegeneration and Memory Loss and Other Symptoms in Women After Menopause

**DOI:** 10.3389/fphar.2021.644103

**Published:** 2021-05-20

**Authors:** Valentina Echeverria, Florencia Echeverria, George E. Barreto, Javier Echeverría, Cristhian Mendoza

**Affiliations:** ^1^ Facultad de Ciencias de la Salud, Universidad San Sebastian, Concepcion, Chile; ^2^ Research and Development Service, Bay Pines VA Healthcare System, Bay Pines, FL, Unites States; ^3^ Department of Biological Sciences, University of Limerick, Limerick, Ireland; ^4^ Health Research Institute, University of Limerick, Limerick, Ireland; ^5^ Departamento de Ciencias del Ambiente, Facultad de Química y Biología, Universidad de Santiago de Chile, Santiago, Chile

**Keywords:** Alzheimer's disease, Parkinson's disease, estrogenic plants, neurodegeneration, neuroprotection, menopause, cognitive impairment

## Abstract

In mammals, sexual hormones such as estrogens play an essential role in maintaining brain homeostasis and function. Estrogen deficit in the brain induces many undesirable symptoms such as learning and memory impairment, sleep and mood disorders, hot flushes, and fatigue. These symptoms are frequent in women who reached menopausal age or have had ovariectomy and in men and women subjected to anti-estrogen therapy. Hormone replacement therapy alleviates menopause symptoms; however, it can increase cardiovascular and cancer diseases. In the search for therapeutic alternatives, medicinal plants and specific synthetic and natural molecules with estrogenic effects have attracted widespread attention between the public and the scientific community. Various plants have been used for centuries to alleviate menstrual and menopause symptoms, such as Cranberry, Ginger, Hops, Milk Thistle, Red clover, Salvia officinalis, Soy, Black cohosh, Turnera diffusa, Ushuva, and Vitex. This review aims to highlight current evidence about estrogenic medicinal plants and their pharmacological effects on cognitive deficits induced by estrogen deficiency during menopause and aging.

## Introduction

Sex hormones such as androgens and estrogens significantly influence behavior (Hamson et al., 2016) and support learning and memory (Andreano and Cahill, 2009; Munro et al., 2012; Colciago et al., 2015; Kerschbaum et al., 2017). The effects of sex hormones on cognition may explain the differences in cognitive abilities among sexes. For example, typically, women have superior verbal memory and are better at recalling and associating words from a spoken list than men (Munro et al., 2012).

Experimental and epidemiological studies suggest that female sex hormones are neuroprotective, preventing cognitive decline during aging. The discovery that brain regions involved in learning and memory, such as the hippocampus and prefrontal cortex, differed in structure and function between sexes suggested that sex hormones played a role in cognition (Kapur et al., 1995; Gasbarri et al., 2012; Pompili et al., 2012). Sex hormones influence aging (Boss et al., 2014) and the incidence, progression, and severity of symptoms in psychiatric disorders (Xing et al., 2013). One interesting potential beneficial effect of estrogens in the brain is to prevent the deterioration of cognitive abilities during aging. Likewise, estrogenic compounds' study revealed a central role of the cerebral estrogen receptors (ER)α and ERβ signaling on cognitive abilities (Baez-Jurado et al., 2019). These receptors have differential expression in the body's tissues and cells. They can have selective effects on different tissues. Most phytoestrogens are full ERβ agonists and weak ligands of the ERα (Gencel et al., 2012; Martinkovich et al., 2014). Estrogenic compounds support cognitive abilities by a mechanism involving the ERα (Tang S.-S. et al., 2018; Mitchnick et al., 2019). This receptor, acting as a transcription factor, controls gene expression in brain regions playing a vital role in learning and memory, such as the hippocampus (Spencer-Segal et al., 2012; Frick et al., 2018; Tang et al., 2019). Also, ERs promote neuroregeneration after traumatic brain injury by stimulating astrocytes' neuroprotective effects (Martin-Jimenez et al., 2019).

**FIGURE 1:**
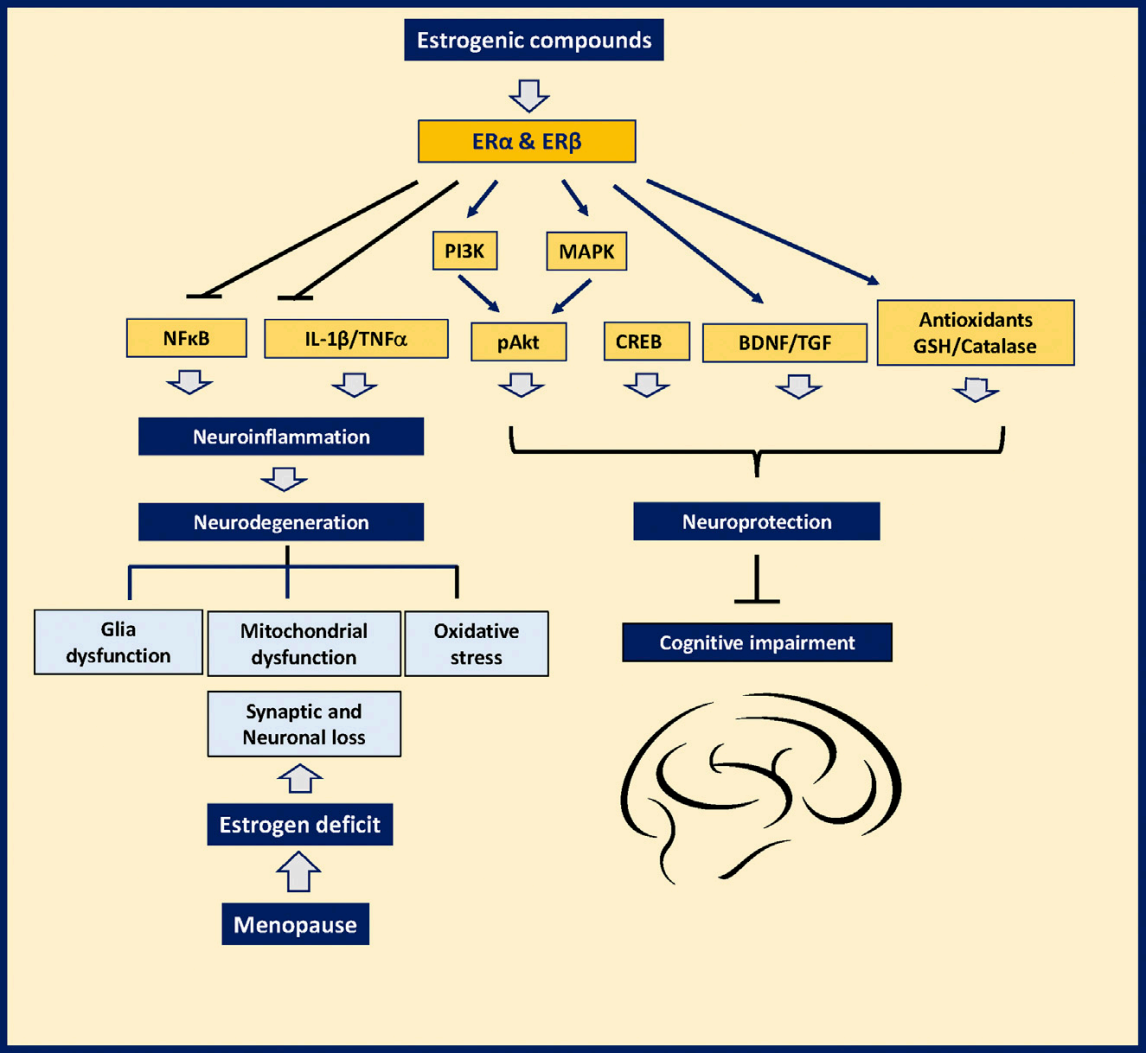
Mechanism of action of estrogenic plants to prevent neurodegeneration and memory loss and other symptoms in women after menopause.

There is abundant evidence on the therapeutic impact of estrogenic compounds and the molecular mechanisms underlying their beneficial effects diminishing inflammation, oxidative stress, and neuronal loss after menopause. These benefits may also account for their positive impact alleviating the cognitive deficits in animal models of two leading types of dementia; Alzheimer’s disease (AD) and Parkinson’s disease (PD) (Figure 1). Tibolone (7-α, 17- α-17-hydroxy-7-methyl-19-norpregn-5 [10]-en-20-ψn-3-one) an estrogenic synthetic drug used as Hormone replacement therapy (HRT), is neuroprotective against the neurotoxic effects of palmitic acid in the brain by protecting astrocytes from inflammation by a mechanism involving the ERβ In experimental models of inflammation induced by a high-fat diet (González-Giraldo et al., 2018; Hidalgo-Lanussa et al., 2018; González-Giraldo et al., 2019; Martin-Jimenez et al., 2019; Hidalgo-Lanussa et al., 2020). On the other hand, the phytoestrogens, also called natural selective estrogen receptor modulators (SERMs), act as agonists or antagonists for ERs. They have different effects on different tissues and cells. The phytoestrogens have structural similarity with 17-β estradiol, showing both agonist and antagonist effects on the ERα and ERβ (Rietjens et al., 2017).

A comprehensive review of pertinent published literature on estrogenic plants using the scientific databases SCOPUS, PubMed, and Science Direct was done. The revision emphasized plants and compounds with beneficial effects against oxidative stress, inflammation, cell viability, and memory loss in cell and *in vivo* studies of AD and PD. Only plants with scientific evidence of estrogenic activity were included.

## Cognitive Deficits During Menopause and Hormone Replacement Therapy

It is estimated that by 2030, the world population of menopausal and postmenopausal women will be 1.2 billion (Borrelli and Ernst, 2008). A systematic review of the literature and random-effect meta-analyses of several clinical studies showed that surgical menopause at any age was associated with a decline in verbal and semantic memory and information processing speed (Georgakis et al., 2019). As discussed in previous reports, the role of estrogen over cognition became more relevant due to the worldwide increase in female life expectancy.

Different ER ligands, including phytoestrogens and synthetic compounds such as tibolone, are under investigation to alleviate or prevent these cognitive deficits. Tibolone has many beneficial effects on the central nervous system (Pinto-Almazan et al., 2017) by mechanisms counteracting the effects of neuroinflammation, oxidative stress, and mitochondrial dysfunction induced by different insults acting on glial cells, including microglia and astrocytes (González-Giraldo et al., 2018; Hidalgo-Lanussa et al., 2018; González-Giraldo et al., 2019; Martin-Jimenez et al., 2019; Osorio et al., 2019; Hidalgo-Lanussa et al., 2020) Tibolone binds to the ERs and has positive effects diminishing the negative consequences of estrogen deficiency on cognition in ovariectomized (OVX) Sprague–Dawley rats by a mechanism involving the enhancement of both the serotoninergic and cholinergic systems (Espinosa-Raya et al., 2012; Pinto-Almazan et al., 2017).

Clinical studies: Considering that menopause arrives on average at 50 years of age, women without intervention can spend more than 30 years in a hypoestrogenic state with several global consequences on cognition and mood (Slaven and Lee, 1997; Shilling et al., 2001). Sex hormones modulate brain plasticity processes involved in high-order cognitive functions such as learning and memory (Frick, 2009; Henderson and Popat, 2011; Frick, 2012; Pompili et al., 2012; Hamson et al., 2016; Korol and Wang, 2018). Clinical studies investigated the correlation between the adult levels of sex hormones and the documented differences in spatial and verbal memory abilities among male and female subjects (Hugdahl et al., 2006).

HRT with estrogens and progestins reduces postmenopausal symptoms but increases the risk of mammary carcinomas and cardiovascular events. For this reason, plant-derived estrogenic compounds mimicking the effect of sex hormones with a better safety profile are highly regarded (Wuttke and Seidlova-Wuttke, 2014).

Although no consensus has been reached (Kocoska-Maras et al., 2011), most clinical studies show that HRT improves cognitive abilities in menopausal women (Sherwin and Grigorova, 2011). One of these studies included 96 non-menopausal control women, 60 women treated with HRT, 56 postmenopausal women, and 41 men. Participants were requested to learn two-word lists. When they learned list 1, they were given either a forget or a remember list 1 cue. When participants had learned list 2 and finished an interference task, they need to write down all items they recalled. Data analysis revealed an effect of sex hormones on learning abilities as follows. Remember-cued control women recalled more list 1 items than HRT users, young men, and postmenopausal women. In forget-cued women, salivary progesterone correlated positively with the recall of items. Salivary 17 β-estradiol did not correlate with the number of recalled items from list one or two in either remember- or forget-cued control women. However, salivary 17 beta-estradiol correlated with item recall in remember-cued postmenopausal women. They concluded that sex hormones do not affect general verbal memory acquisition or consolidation but modulated cue-specific learning and memory recall (Kerschbaum et al., 2017). Another study showed that HRT with estrogen plus progesterone improved speed cognitive processing in women carrying the Apolipoprotein E (APOE) polymorphisms epsilon (ε) 2/ε3, and ε4. However, women receiving HRT but carrying e3/e3 alleles showed worse verbal memory scores in the Montreal Cognitive Assessment test than control subjects (Bojar et al., 2013). The study of the relationship between endogenous levels of sex hormones (estradiol, estrone, progesterone, and free testosterone) and cognitive abilities (verbal episodic memory, executive functions) showed that progesterone concentrations positively correlated with verbal memory and global cognition (Henderson et al., 2013). They also found that the level of sex hormone-binding globulin positively correlated with verbal memory performance (Henderson et al., 2013).

Various studies revealed that estrogen and progesterone positively influence verbal memory in females, and this and other cognitive skills in postmenopausal women (Keenan et al., 2001; Maki and Hogervorst, 2003; Hogervorst et al., 2009; Brown, 2013; Doty et al., 2015; Pinto-Almazan et al., 2017; Moradi et al., 2018). Nevertheless, clinical studies by the Women's Health Initiative showed that women who initiated estrogen treatment alone or combined with progestin at older ages (65–79 years of age) presented a higher risk of cognitive decline and dementia (Rocca et al., 2011). This discrepancy can be explained by the window of opportunity hypothesis, suggesting that estrogen's effects will vary depending on age, cause, and stage of menopause. It seems that in women, estrogen treatment is neuroprotective when started early after menopause (50–60 years of age). The authors recommend that women younger than 51 years of age with premature natural menopause should benefit from HRT (Rocca et al., 2011). Based on this evidence, it is reasonable to postulate that estrogen deficiency can be a crucial factor affecting brain function after menopause.

## Estrogenic Plants

Estrogenic plants have been used from ancient times to diminish the symptoms of menopause, such as hot flushes, cognitive impairment, and depression. The most commonly used are the Black cohosh (*Actaea racemosa* L) (Wuttke et al., 2014), Ginger (*Zingiber officinale* Roscoe)*,* Hops (*Humulus lupulus* L.)*,* Milk thistle (*Silybum marianum*)*,* golden root (*Rhodiola rosea* L.)*,* Red clover (*Trifolium pratense* L)*,* Sage (*Salvia officinalis* L)*,* Soy (*Glycine max* L)*,* Damiana (*Turnera diffusa* Willd. ex Schult)*,* Ushuva (*Physalis peruviana* L)*,* and vitex (*Vitex agnus-castus* L)*.* However, many more have been investigated (Table 1).

**TABLE 1:** Traditional medicinal plants with estrogenic activity.

	Scientific name	Family	Common name	Extract/compound	Model	Effect	References
	*Achillea millefolium* L	Asteraceae	Yarrow	Methanolic extract	MCF-7 cells	Stimulation of the ER *ß*	Innocenti et al. (2007)
	*Adenophora stricta *Miq	Campanulaceae	Sha shen and ladybells	Methanolic extract	Recombinant yeast system	EA	Kim et al. (2008)
	*Anemarrhena asphodeloides *Bunge	Asparagaceae	Zhi mu	Methanolic extract	Recombinant yeast system	EA	Kim et al. (2008)
	*Angelica sinensis *(Oliv.) Diels	Apiaceae	Dong quai, dang gui	Ferulic acid and Z-Ligustilide	OVX rat	EA	Liu et al. (2001)
							Circosta et al. (2006)
	*Artemisia vulgaris *L	Asteraceae	Mugwort	Eriodictyol and apigenin	*Saccharomyces cerevisiae* strain BJ3505	EA	Lee et al. (1998)
	*Asplenium trichomanes *L	Aspleniaceae	Maidenhair spleenwort, tie jiao jue	Leaves infusion, decoction, and methanol extract	MCF7/EREluc cell line, which expresses endogenous ERα, and SK-NBE cells transiently transfected with the ERα and ERβ	Stimulation of ERα and ERβ	Dall'Acqua et al. (2009)
	*Astragalus mongholicus* Bunge. syn. *Astragalus membranaceus*	Fabaceae	Mongolian milk, huang qi	Decoction	MCF-7 cells	Activation of ERE	Gong et al. (2016)
	*Elettaria cardamomum *L	Zingiberaceae	Cardamon	Methanolic extract	MCF-7 breast cancer cells using the E-Screen bioassay	EA	Real et al. (2015)
	*Cocos nucifera *L	Arecaceae	Coconut palm	Juice	OVX rat	EA on the progression of AD-like pathology	Radenahmad et al. (2009)
	*Cullen corylifolium *(L.) Medik	Fabaceae	Bu gu zhi, cule	Ethanolic extract	*Saccharomyces cerevisiae* ER + LYS 8127, MCF-7 cells	Stimulation of ERβ and ERα	Lim et al. (2011)
	*Cynomorium coccineum *subsp. *songaricum* (Rupr.) J. Léonard	Cynomoriaceae	Hongo de Malta and jopo de lobo	Ethanolic extract	Recombinant yeast system	EA	Zhang et al. (2005)
	*Curcuma aromatica *Salisb	Zingiberaceae	Yu jin, wild turmeric	Rhizome extract	Recombinant yeast system	EA	Kang et al. (2006)
	*Dunbaria villosa *(Thunb.) Makino	Fabaceae	Ye bian dou	Methanolic extract	Ishikawa cells	EA	Yoo et al. (2005)
	*Epimedium brevicornu *Maxim	Berberidaceae	Yin yang huo, horney goat weed	Ethanolic extract	Recombinant yeast system	EA	Zhang et al. (2005)
	*Glycine max* (L.) Merr	Fabaceae	Soybean, da dou, soya	Methanolic extract/Biochanin A, genistein, daidzein, formononetin, and glycitein	MCF-7 cells	EA, binding to ERα and ERβ and the PR	Boue et al. (2003)
	*Glycyrrhiza uralensis *Fisch	Fabaceae	Gan cao, Chinese liquorice	Ethanolic extract	Recombinant yeast system	EA	Kang et al. (2006)
	*Glycyrrhiza glabra *L	Fabaceae	Licorice, yang gan cao, regaliz	Ethyl acetate extract from licorice root	Recombinant yeast system	EA toward either one or both ERα and ERβ	Simons et al. (2011)
	*Humulus lupulus *L	Cannabaceae	Hops, pi jiu hua	Methanolic extract	Ishikawa cells	Competitive binding to ERα and ERβ	Liu et al. (2001)
	*Hylodesmum podocarpum* subsp. *oxyphyllum* (DC.) H.Ohashi and R.R.Mill. syn. Desmodium oxyphyllum	Fabaceae	Jian ye chang bing Shan ma huang, tick-trefoils	Methanolic extract	Ishikawa cells	EA	Yoo et al. (2005)
	*Iris domestica* (L.) Goldblatt & Mabb. syn. *Belamcanda chinensis*	Iridaceae	Vanilla domestica, blackberry lilly	Ethanolic extract	Recombinant yeast system	EA	Zhang et al. (2005)
	*Kummerowia striata* (Thunb.) Schindl	Fabaceae	Ji yan cao, Japanese clover	Methanolic extract	Ishikawa cells	EA	(Yoo et al., 2005)
	*Lespedeza bicolor *Turcz	Fabaceae	Shrub bush-clover, bicolored lespedeza, shrub lespedeza, hu zhi zi	Methanolic extract	Ishikawa cells	EA	Yoo et al. (2005)
	*Lycopus cavaleriei* H. Lév. syn. *Lycopus ramosissimus*	Lamiaceae	Xiao ye di su, ko-shiro-ne	Dichloromethane extraction	Recombinant yeast system	EA	Kim et al. (2008)
							Kang et al. (2006)
	*Maackia amurensis *Rupr	Fabaceae	Amur maackia, chao xian huai	Methanolic extract	Ishikawa cells	EA	Yoo et al. (2005)
	*Maackia floribunda* (Miq.) Takeda syn. *Maackia fauriei*	Fabaceae	Hanemi-inuenju, duo hua ma an shu	Methanolic extract	Ishikawa cells	EA	Yoo et al. (2005)
	*Medicago sativa *L	Fabaceae	Alfalfa sprout, zi mu xu	Methanolic extract/Biochanin A, genistein, daidzein, formononetin, and glycitein	MCF-7 cells	EA-binding to the ERα and ERβ and PR	Boue et al. (2003)
	*Panax ginseng* C.A. Mey. and *Panax quinquefolius *L	Araliaceae	Asian ginseng and north american ginseng, respectively	Methanolic extract	Ishikawa cells	Competitive binding to ERα and ERβ	Liu et al. (2001)
	*Phaseolus vulgaris *L	Fabaceae	Green bean Red kidney bean	Methanolic extract	MCF-7 cells	Estrogenic and antiestrogenic activities	Boue et al. (2011)
	*Prunus persica* (L.) Batsch	Rosaceae	Peach, melocotón	Methanolic extract	Recombinant yeast system	EA	Kang et al. (2006)
	*Pueraria montana* var. lobata (Willd.)	Fabaceae	Ge ma mu, kudzu root	Methanolic extract	Recombinant yeast systemcultured MCF-7 cells ishikawa cells	EA-binding to the ERα ERβ and PR	Kang et al. (2006)
							Boue et al. (2003), Yoo et al. (2005)
	*Reynoutria japonica* Houtt. syn. *Fallopia japonica* and *Polygonum cuspidatum*	Polygonaceae	Hu zhang	Ethanolic extract	Recombinant yeast system	EA	Zhang et al. (2005)
	*Reynoutria multiflora*(Thunb) Moldenke syn *Polygonum multiflorum *(Thunb)	Polygonaceae	Chinese knotweed, He shou Wu, fo-ti	Ethanolic extract	Recombinant yeast system	EA	Zhang et al. (2005)
							Kang et al. (2006)
	*Rheum palmatum *L	Polygonaceae	Chinese rhubard, zhang ye da huang	Ethanolic extract	Recombinant yeast system	EA	Zhang et al. (2005)
							Kang et al. (2008)
	*Rhodiola rosea *L	Crassulaceae	Golden root, rose root, arctic root, orpin rosea, hong jing tian	Extract (flavonoids)	Molecular docking	EA (ERα and ERβ)	Powers and Setzer (2015)
	*Salvia officinalis *L	Lamiaceae	Sage, garden sage, salvia	Aqueous-ethanolic extract	Cell line T7D-Kbluc	Estrogenicity (ERLUX assay)	Rahte et al. (2013)
	*Salvia lavandulifolia *Vahl	Lamiaceae	Spanish sage	Ethanolic extract	Recombinant yeast system	EA	Perry et al. (2001)
	*Scutellaria baicalensis *Georgi	Lamiaceae	Huang qin, Chinese skullcap	Ethanolic extract	Recombinant yeast system	EA	Zhang et al. (2005)
	*Senna obtusifolia* (L.) H.S. Irwin & Barneby. syn *Cassia obtusifolia *L	Fabaceae	American sicklepod	Ethanolic extract	Recombinant yeast system	EA	Zhang et al. (2005)
	*Sophora flavescens *Aiton	Fabaceae	Ku shen	Ethanolic extract	Recombinant yeast system and Ishikawa cells	EA	Kang et al. (2006)
							Yoo et al. (2005)
	*Trifolium pratense *L	Fabaceae	Red clover, hong che zhou cao	Methanolic extract/Biochanin A, genistein, daidzein, formononetin, and glycitein	MCF-7 cells	EA binding to the ERα and ERβ and PR	Boue et al. (2003)
	*Turnera diffusa *Willd	Passifloraceae	Damiana, oreganillo, mexican tea	Methanolic extract	Yeast estrogen screen (YES) assay	EA	Zhao et al. (2008)
	*Vitex agnus-castus *L	Lamiaceae	Vitex, chaste tree, chasteberry	Methanolic extract	Ishikawa cells	Competitive binding to ERα and ERβ	Liu et al. (2001), Liu et al. (2004)
	*Vigna radiata *(L.)	Fabaceae	Lu dou, mung bean	Methanolic extrac Biochanin A, genistein, daidzein, formononetin, and glycitein	MCF-7 cells	EA binding to the ERα, ERβ, and PR	Boue et al. (2003)
	*Wurfbainia villosa* var. *xanthioides*, syn. *Amomum xanthioides* (Wall ex Baker) Skornick. and A.D. Poulsen (syn, *Amomum xanthioides *Wallich)	Zingiberaceae	Malabar cardamom	Methanolic extract	Recombinant yeast system	EA	Kim et al. (2008)
	*Zingiber officinale *Roscoe	Zingiberaceae	Jiang, ginger	Methanolic extract	Recombinant yeast system	EA	Kim et al. (2008)
							Kang et al. (2006)

The positive effects of sex hormones on cognition encouraged the study of phytoestrogens present in medicinal plants to alleviate the cognitive deficits during menopause. A modern study used molecular docking investigated more than 500 phytochemicals from 17 herbal supplements sold in the United States to identify potential estrogenic or anti-estrogens herbal compounds. Each compound was docked with the known structures of the ERα and ERβ. The results revealed numerous compounds that strongly docked with the ER, including *Echinacea,* milk thistle (*Silybum marianum*)*, GB, Sambucus* nigra*,* chaste tree (*Vitex agnus-castus*)*,* fenugreek (*Trigonella foenum-graecum*)*, Rhodiola rosea,* Licorice (*Glycyrrhiza glabra*)*,* wild yam (*Dioscorea villosa*)*,* black cohosh (*Actaea racemosa*)*,* muira puama (*Ptychopetalum olacoides or P. uncinatum*), red clover (*Trifolium pratense*)*,* damiana (*Turnera aphrodisiaca or T. diffusa*)*,* and dong quai (*Angelica sinensis*). Also, some men's herbal supplements showed robust docking to the ER: *GB*, *gotu kola* (*Centella asiatica*), muira puama (*Ptychopetalum olacoides or P. uncinatum*), and *Tribulus terrestris.* The authors concluded that almost all popular herbal supplements contain phytochemical components that may bind and modulate hER, causing unwanted effects (Powers and Setzer, 2015).

### Phytoestrogens

Phytoestrogens including flavonoids, isoflavonoids, stilbenes, lignans, ginsenosides, tetrahydrofurandiol, chalcones, and coumestans, and are commonly found in fruits and other parts of various medicinal plants (Slavin and Lloyd, 2012). Various traditional plants to alleviate the effects of menopause contain phytoestrogens (Lorand et al., 2010). In addition to their use to counteract menopause symptoms (Rowe and Baber, 2021), phytoestrogens for their antioxidant and anti-inflammatory properties may have many other therapeutic uses including being considered anti-cancer drugs (Ardito et al., 2018; Basu and Maier, 2018; Petrine and Del Bianco-Borges, 2021), regulators of cholesterol metabolism, hepatoprotective (Alipour and Karimi-Sales, 2020) and cardioprotective compounds (Asokan Shibu et al., 2017). These properties have a broad impact on human health under normal and pathological states in aging, including beneficial effects on cognitive abilities (Thaung Zaw et al., 2017; Dominguez and Barbagallo, 2018; Hussain et al., 2018). Phytoestrogens at low doses have little or no effects on climacteric complaints, but they mimic estrogen effects at high doses.

Phytoestrogens display higher selectivity for specific ERs permitting more organ-specific actions than estrogen. For example, the flavonoids catechin, rutin, daidzein, luteolin, naringenin, and genistein are present in powder extracts of *Lespedeza bicolor*. In behavioral experiments, *L. bicolor*’s extracts (25 and 50 mg/kg) improved the cognitive impairment induced by intracerebroventricular injection of Aβ_25-35_ in several cognitive tests, including the novel object recognition. These plant extracts enhanced the endogenous antioxidant system increasing the glutathione (GSH) expression and inhibiting the hippocampus’s acetylcholinesterase activities. Also, *L. bicolor* increased the expression of the brain-derived neurotrophic factor (BDNF), and phospho-Akt, extracellular signal-regulated kinase (ERK), and cAMP response element (CRE) expression that is reduced in the hippocampus by Aβ. In conclusion, *L. bicolor* exerts a memory-enhancing effect in an Aβ-induced mouse model of AD-like pathology (Ko et al., 2019). Phytoestrogen-primarily the isoflavones genistein, daidzein and coumestrol, stemming from soy (*Glycine max*) or red clover (*Trifolium pratense*)—were suggested to have the desired but not the undesired effects of estrogens.

However, the results of the most recent placebo-controlled studies question the beneficial effects of phytoestrogens. Nevertheless, when taken at the time of puberty, phytoestrogens seem to prevent mammary cancer in adulthood (Seidlova-Wuttke et al., 2013).

## Estrogenic Plants for Menopause

### *Astragalus mongholicus* Bunge.

*Astragalus mongholicus* Bunge. (syn.* Astragalus membranaceus*) (Fabaceae), commonly named Mongolian milk, is used as a constituent of the traditional Chinese medicine (TCM) named Danggui Buxue Tang (DBT). DBT is an herbal decoction containing *A. mongholicus* and *Angelica sinensis* roots (5:1 w/w) that is employed to diminish menopausal symptoms. Interestingly, calycosin, the main flavonoid in *A. mongholicus*, has a structure similar to β-estradiol. Based on this evidence, Gong investigated DBT and Calycosin's ability to activate the estrogen-responsive element (ERE) in cultured MCF-7 cells (Gong et al., 2016). DBT has an EA that can be enhanced 2–5 times by Calycosin that did not show EA by itself. A derivative from DBT, the herbal recipe-RRF that is composed of *Radix Astragali* (root of *A. mongholicus*), Radix *Angelicae sinensis* (root of *A. sinensis*), and *Folium Epimedii* (leaves of *Epimedium brevicomum *Maxim.). RRF has shown a remarkable protective effect in OVX rats and represents a promising candidate for treating perimenopausal disorders (Xie et al., 2012). The experiments consisted of giving daily oral treatments with vehicle (Sham and OVX group), RRF (141, 282, and 564 mg/kg), and conjugated equine estrogens (0.1 mg/kg) for 16 weeks. They analyzed bone mineral density (BMD) assay and oxidative stress. The results showed that repeated administration of RRF attenuated osteoporosis by elevating the BMD levels of the total body and arrest bone trabeculae degradation. 3) RRF exposure decreased serum levels of constituent MDA and increased endogenous Superoxide dismutase (SOD) activity (Xie et al., 2012).

### *Cynomorium coccineum* subsp. *songaricum* (Rupr.) J.Léonard

*Cynomorium coccineum* subsp. *songaricum* (Rupr.) J.Léonard (Cynomoriaceae) is a estrogenic medicinal plant is commonly known as Hongo de malta, and Jopo de Lobo grows in Mediterranean countries. Ethanolic *C. coccineum* has an estrogenic RP of 1.78 × 10^–4^ (Zhang et al., 2005). Recent studies reported that *C. coccineum* was antidepressant in perimenopausal rats (Miao et al., 2017) and reduced memory impairment in OVX rats (Tian et al., 2019). In this plant, phytoestrogen- and phytoandrogen-like effects have been detected (Wang et al., 2017; Tao et al., 2019) and various type of compounds, including eight triterpenoids, six flavonoids, four fatty acids, eight phenolic acids, one anthraquinone, one nucleoside, and one sterol have been identified (Li X. et al., 2020).

### *Vitex agnus-castus* L.

*Vitex agnus-castus* L. (Lamiaceae), also known as chaste-tree berry, is used to treat menopause symptoms and menstrual dysphoria. The estrogenic effect of extracts of chaste-tree berry has been investigated using cultured cells (Liu et al., 2001; Liu et al., 2004). These studies reported that methanol extracts of this plant increased the mRNA expression of ERβ and PR in the ER + hormone-dependent T47D: A18 cells (breast cancer cell line) and Ishikawa cells (endometrial cancer cell line). The analysis of the extracts using an ER binding bioassay revealed the presence of linoleic acid, a lipid with presumed EA, in the fruits. *Vitex agnus-castus* L (Liu et al., 2001; Liu et al., 2004). *Chasteberry*, like other estrogenic plant, induced the expression of the estrogen-inducible gene pS2 in the breast cancer cell line S30 (Liu et al., 2001).

### *Epimedium brevicornu* Maxim.

*Epimedium brevicornu* Maxim. (Berberidaceae) is a TCM commonly known as Horney goat weed. A study investigating the effect of Ethanolic extract of *E. brevicornu* showed an EA (RP = 2.30 × 10–4) (Zhang et al., 2005). Besides, the investigation of the effect of different plants of the TCM on BMD found that treatment with extracts of *E. brevicornu* stimulated cyclic AMP response element (CRE) and the expression of osteoblastic markers such as Runx2 and Bmp4 in MC3T3-E1 cells (Rebhun et al., 2019). Also, *Astragalus onobrychis* extracts inhibited the Interleukin (IL)-1β-induced activation of the nuclear factor kappa B (NFκB) and a mix of these plants prevented the loss of BMD with efficacy comparable to estradiol in OVX rats (Rebhun et al., 2019).

### *Glycine max* (L.) Merr

*Glycine max* (L.) Merr (Fabaceae) generally identified as soybean, contains several phenolic compounds with EA, including biochanin A, genistein, daidzein, formononetin, and glycitein. These compounds activate both the ERα and ERβ and bind to the progesterone receptor (PR) and androgen receptor (AR). For example, Biochanin A, daidzein, genistein, and formononetin bind to the PR and AR at concentrations of 0.39–110 mM (Beck et al., 2003). A study showed that Soybean extracts exhibited selectivity for the ERβ and induced a higher cell proliferation level than estradiol (Boue et al., 2003). The increase in cell proliferation was suppressed by the estrogen antagonist, ICI 182,780, suggesting that the ER was involved (Boue et al., 2003). A double-blind, randomized, placebo-controlled, 12 weeks trial evaluated the effect of high-dose isoflavones on quality of life (QOL), cognition, plasma levels of lipoproteins, and androgens in postmenopausal women attended at a tertiary care center in the United States (Basaria et al., 2009). Ninety-three healthy, ambulatory, and postmenopausal women (mean age 56 years) were randomly assigned to receive soy protein (20g-160 mg of total isoflavones) or taste-matched placebo (20 g whole-milk protein), with 84 women (90%) completed the study. QOL was judged by the Menopause-specific Quality of Life (MENQOL) questionnaire. Total, free, and bioavailable testosterone, gonadotropins, Sex Hormone Binding Globulin (SHBG), and fasting lipids were measured. The study found significant improvements in all QOL subscales (vasomotor, psychosexual, physical, and sexual) among the women treated with isoflavones, while no changes were seen in the placebo group (Basaria et al., 2009). No significant changes in cognition, serum androgens, or plasma lipids induced by isoflavones were observed. Total testosterone and HDL levels were significantly lower in the people treated with isoflavones when compared to the placebo group. The timing of isoflavone supplementation with regards to the onset of menopause appears to be important. The authors concluded that high doses of isoflavones are associated with improved QOL among women at the early stages of menopause, indicating that isoflavones as alternatives to estrogen therapy may be useful and safe to relieve menopausal symptoms (Basaria et al., 2009).

### *Angelica sinensis* (Oliv.)

*Angelica sinensis* (Oliv.) (Apiaceae) commonly known as Dong quai, and it is one of the most used medicinal plants. It is prescribed for gastrointestinal, vascular, mental, and gynecological disorders (Giacomelli et al., 2017). The primary components identified in the plant extracts, Ferulic acid, Z-Ligustilide, and polysaccharides, showed weak ER binding and pS2 mRNA induction in the cells (Liu et al., 2001). The administration of standardized ethanol extracts of *A. senensis* to OVX rats stimulated the uterine histoarchitecture, induced cornification of the vaginal epithelium establishing an epidermal barrier, and reduced luteinizing hormone (LH) concentration in serum. Moreover, plant extracts' administration provoked a significant modification of the vaginal smear in 67% of female control rats. Treated rats showed a prolonged estrus stage with a transient interruption of their regular cyclicity (Circosta et al., 2006). All this evidence suggests estrogenic effects for this plant.

### *Rhodiola rosea* L.

*Rhodiola rosea* L. (Crassulaceae) is also known as golden root, rose root, roseroot, Aaron's rod, Arctic root, king's crown, lignum rhodium, and orpin rosea. *R. rosea* has been employed in Russia for a half-century, and ethanolic extract of this plant (40% v/v) are used to relieve fatigue and increase attention, memory, and work productivity. The typical dose recommended is 5–40 drops 2–3 times a day, 15–30 min before eating for 10–60 days. In Denmark, *R. rosea* is registered as a botanical drug and widely used in Scandinavian countries to increase cognitive abilities under stressful conditions. A phytochemistry analysis of *R. rosea* has revealed six distinct groups of compounds present in the root: Phenylpropanoids: rosavin, rosin, rosarin (specific to *R. rosea*); Phenylethanol derivatives: salidroside (rhodioloside), tyrosol; Flavonoids: rodiolin, rodionin, rhodiosin, acetylrodalgin, tricin; Monoterpernes: rosiridol, rosaridin; Triterpenes: daucosterol, beta-sitosterol; Phenolic acids: chlorogenic and hydroxycinnamic, and gallic acids (Elameen et al., 2010; Elameen et al., 2020; Shikov et al., 2020). *R. rosea* is a selective ER modulator, anti-inflammatory, and NFκB inhibitor, as well as an inducer of the endothelial nitric oxide synthase (NOS) that protects osteoblasts from hydrogen peroxide toxicity (Gerbarg and Brown, 2016).

### *Anemarrhena asphodeloides* Bunge

*Anemarrhena asphodeloides* Bunge (Asparagaceae), commonly named zhi mu and rhizome, is native to China, Korea, and Mongolia is a TCM. In Korea, *A. asphodeloides* B is employed as antipyretic, diuretic, sedative, and antitussive medicine (Lee et al., 2018). The study of ethanol extracts of *A. asphodeloides* B revealed anti-osteoporotic effects, inhibiting osteoclastogenesis and reducing inflammation (Kim et al., 2017). Dichloromethane fractions of *A. asphodeloides* B showed EA with a RP of 1.9 × 10^–4^ (RP of Estradiol (E2) was 1.0) (Kim et al., 2008). Methanolic extract (0.1 mg/ml) showed moderate EA (Kang et al., 2006).

### *Salvia officinalis* L.

*Salvia officinalis* L. (Lamiaceae), also known as sage, is one of the most abundant species of *Salvia* (>500 species). These flowering plants, native to the Mediterranean countries, have been used as traditional medicines for centuries. *S. officinalis* contains phenolic diterpenoids with antioxidants and anti-inflammatory activities such as carnosic acid and carnosol (Abdellatif et al., 2020). *S. officinalis* has EA and is used to control hot flashes. A study investigating an aqueous-ethanolic extract of this plant discovered EA in the ERLUX assay with an EC_50_ = 64 mg/ml (Rahte et al., 2013). Fractionation of the EA in the extracts revealed that luteolin-7-O-glucuronide (EC_50_ = 159 mg/ml) was the active estrogenic compound. The authors concluded that the positive effect of *S. officinalis* on hot flashes must be due to the effect of estrogenic flavonoids present in *Salvia* (Rahte et al., 2013).

### *Scutellaria baicalensis* Georgi

*Scutellaria baicalensis* Georgi (Lamiaceae) is also known as Baickal Skullcap and Huang Qin. An early study found that ethanolic extracts of *S. baicalensis* have EA (RP of 8.77 × 10^–5^) (Zhang et al., 2005). Additional studies have investigated *S. baicalensis* alone or as part of herbal mixtures to treat health conditions related to hormone dysregulation such as tumors (Murashima et al., 2009), endometriosis (Ferella et al., 2018), and prostate cancer (Hsieh and Wu, 2002). In a prostate cancer study, LNCaP cells were treated with ethanol extracts of the herbal mix PC-SPES or their components. Cells treated with PC-SPES showed a 70–80% reduction in cell growth and viability. *S. baicalensis* extracts halted cell growth by a 66.5% (Hsieh and Wu, 2002).

### *Actaea racemosa* L.

*Actaea racemosa* L. (syn. *Cimicifuga racemosa* (L.) Nutt.) (Ranunculaceae) is also known as black cohosh. Extracts of the rhizome of black cohosh have shown no having estrogenic effects on mammary cancer cells *in vitro* and on mammary gland and uterine histology in OVX rats. However, in those rats, the extract *A. racemosa* L. BNO1055 reduced hot flushes and the occurrence of osteoporosis (Seidlova-Wuttke et al., 2003). Further reports from same author suggested that *Actaea racemosa* L. extracts at low doses are adequate to ameliorate climacteric complaints without adverse estrogenic effects (Wuttke et al., 2014).

Clinical studies: In postmenopausal women, compared to placebo *Actaea racemosa* L. BNO 1055 in a meaningful manner reduced menopause symptoms similarly to conjugated estrogens. Similar data were published for other European *Actaea racemosa* L. preparations, whereas 2 US American preparations were ineffective, most likely due to the doses or purity of the preparations used. The European studies did not report *Actaea racemosa* L. effects in the uterus nor mammary glands.

A recent study evaluated the efficacy on climacteric problems and safety of a combination of plant extracts (soy isoflavone, black cohosh, chasteberry, and evening primrose oil extracts) in post-menopausal women (Rattanatantikul et al., 2020). The study consisted of a randomized, double-blinded, placebo-controlled trial. Post-menopausal women of 45–60 years old were randomly assigned to either treatment (*n* = 50) or placebo group (*n* = 51). Herb extracts and placebo were administered to participants, and at baseline and after 6 and 12 weeks of treatments, symptoms of menopause, endocrine profile, and blood chemistry were assessed. Herbs' combination significantly reduced hot flushes and sweating, sleep problems, depressed mood, and irritability symptoms compared with the placebo group. There were no significant differences in hormonal levels between the test and placebo groups; however, the C-reactive protein levels were found significantly decreased. Moreover, serum LDL-C and triglyceride levels were significantly lower than baseline levels in the treatment group at 6- and 12 weeks timepoints. No adverse effects were reported during the trial. These data indicate that this combination of four medicinal herbs effectively and safely improves menopausal symptoms, as well as general health indicators, in post-menopausal women (Rattanatantikul et al., 2020).

## Estrogenic Plants and Compounds With Potential Effects Against Alzheimer's Disease

Some estrogenic plants have been investigated as therapeutic tools to prevent memory loss after menopause and reducing the risk of developing the most frequent neurodegenerative conditions. AD is a neurodegenerative disease characterized by cholinergic deficits, neuronal loss, glial dysfunction (Echeverria et al., 2009), intracellular and extracellular accumulation of aggregated forms of Aβ, and the appearance of intraneuronal neurofibrillary tangles of hyperphosphorylated Tau in the brain (McGeer, 1986; Mann, 1989; Masdeu et al., 2005). The main risk factors involve traumatic brain injury (Gardner and Yaffe, 2015; Daulatzai, 2017), posttraumatic stress disorder (PTSD) (Tsolaki et al., 2009; Meziab et al., 2014; Ritchie et al., 2019), diabetes (Chen and Zhong, 2013; Boccardi et al., 2019; Jash et al., 2020), and age (Simpson et al., 2010; Bartzokis, 2011; Bove et al., 2014). These abnormalities occur with neuroinflammation and oxidative stress that orchestrate a cascade of events leading to cognitive decline, gliosis, and synaptic loss. This idea is endorsed by studies showing that inhibition of pro-inflammatory cascades improves cognitive abilities in transgenic AD mice. Indeed, the posttreatment of AD mice with the Raf inhibitor Sorafenib inhibited the expression of several downstream inflammatory factors such as NFkB and nitric oxide synthase (Echeverria et al., 2009). Medicinal plants having anti-inflammatory effects can be of use in preventing AD after menopause. Women are more affected by AD, indicating that sex hormones are involved in the disease progression (Vina and Lloret, 2010). Conditions affecting estrogen production, such as premature ovarian insufficiency in women less than 40 years of age, are associated with neurological dysfunction and an increased risk of dementia (Soni and Hogervorst, 2014).

Preclinical studies suggest a role for estrogenic compounds preventing AD (Saunders-Pullman, 2003) (Morale et al., 2006) (Morissette et al., 2008) (Siani et al., 2017) and other neurological conditions (Hugdahl et al., 2006; Pompili et al., 2012). The putative role of female hormones in the development of dementia may explain the finding that women with a late menopausal arrival (>53 years of age) have a decreased risk of AD. It has been proposed that brain differences in the cholinergic system among males and females may underlie the differences in response to acetylcholinesterase inhibitors (AChEI) and the rate of progression of AD between sexes, with a higher benefit of AChEI treatment delaying the progression of the disease in men than women (Giacobini and Pepeu, 2018). Estrogenic compounds are neuroprotective against Aβ toxicity *in vitro* (Svensson and Nordberg, 1999; Szego et al., 2011; Xing et al., 2011; Bagheri et al., 2012; Wu et al., 2012; Napolitano et al., 2014; Costa et al., 2016; Li et al., 2017; You et al., 2017; Yang et al., 2019) as well as *in vivo* models of AD (Bagheri et al., 2011; Limon et al., 2012; Pompili et al., 2012; Zhao et al., 2013). In one of these preclinical studies, to investigate the effect of estrogen on the cholinergic deficit in AD, different Aβ assemblies were injected in the nucleus basalis magnocellular (NBM) of sham control and OVX mice, and the viability of cholinergic cells was measured (Szego et al., 2011). The results showed that Aβ oligomers were the most neurotoxic aggregated species and estrogen treatment before Aβo infusion prevented cholinergic neuronal loss (Szego et al., 2011). Furthermore, a proteomic analysis of the NBM revealed significant differences among Aβ-treated mice and control mice in the expression of the mitochondrial factors DJ-1, NADH ubiquinone oxidoreductase, ATP synthase, phosphatidyl-ethanolamine-binding protein 1, protein phosphatase (PP) 2A, and dimethyl-arginine dimethyl-aminohydrolase 1 (Szego et al., 2011). Besides, Aβ inhibited MAPK signaling and stimulated NOS activity. The observed increase in the activities of PP1 and PP2A may explain the inhibition of MAPK signaling in the neocortex. Impressively, pretreatment with estrogen prevented most of these proteome changes (Szego et al., 2011). A few years ago, the role of the ERα, a transcriptional factor involved in mitochondrial and energy metabolism, was investigated in a transgenic (Tg) model of AD expressing the amyloid β precursor protein (APP) and presenilin 1 (PS1) containing familial AD (FAD) mutations (Tg APP/PS1 mice). They found that the expression of the ERα was progressively reduced in the brain of AD mice. More importantly, HEK293 cells stably expressing the human APP, and ERα showed reduced production of Aβ peptides, decreased Tau phosphorylation, and reduced activity of a critical Tau kinase *in vivo*, the glycogen synthase kinase 3β (GSK3β) (Tang Y. et al., 2018). Coherent with the preclinical evidence, the results of several clinical studies suggest that estrogen may prevent cognitive decline and improve cognitive function in postmenopausal women (Sherwin, 2012; Brown, 2013; Doty et al., 2015). Estrogen or HRT would be an adequate preventative treatment to reduce the risk of dementia in women with a low risk of cardiovascular disease and a heightened risk of developing dementia, For example, a person with a family history of AD (Loucks and Berga, 2009). However, some studies indicated that the effect of estrogen could also depend on the age of the person treated as conjugated equine estrogens plus medroxyprogesterone acetate seems to augment the risk of dementia in women over 65 years of age (Sano et al., 2008; Maki and Henderson, 2012).

Medicinal plants have been used throughout the history of humanity to improve mental abilities. The connection between the estrogenic neuroprotective effect of medicinal plants and their role against neurodegeneration has recently been investigated. One of these investigations by Zhao et al. described a phytoestrogen formulation named Phyto-β-SERM, which exhibited a higher binding selectivity (83 times) for ERβ than ERα. Phyto-β-SERM has central estrogenic neuroprotective effects without inducing feminizing effects over the body. Furthermore, chronic exposure to Phyto-β-SERM prevented menopause symptoms in mice. Additionally, nine-month treatment with Phyto-β-SERM prolonged survival improved spatial recognition memory and reduced cerebral amyloid-β plaque deposition in female triple-transgenic AD mice. A gene expression analysis of AD mouse brains showed that phytoestrogens were neuroprotective by a mechanism involving the crosstalk between the ERβ and glycogen synthase kinase 3 (GSK3β). This evidence supports the therapeutic value of phytoestrogen for preventing AD (Zhao et al., 2013). Various studies investigated whether there were changes in estrogen/ERα expression on AD. Since ERα gene expression is regulated by methylation (Frick, 2015), a recent clinical study examined whether the methylation level of the promoter of the ERα gene and its protein expression correlated with changes in cognitive function and quality of daily living in AD. The QOL of the participants was tested with the QOL- AD scale and their cognitive abilities were assessed using both the Mini-Mental State Examination (MMSE) and the activities of daily living (ADL) tests. The methylation status and level of expression of the ERα gene were determined in the peripheral blood of 132 patients with AD and 135 healthy control subjects (Li et al., 2019). This study showed that a higher level of methylation of the ERα gene promoter correlated with a decreased ERα expression and reduced cognitive abilities in AD patients (Li et al., 2019). This evidence supports the view that lower estrogenic signaling correlates with cognitive impairment in AD. While most phytoestrogens act through ERα and ERβ, some of their biological actions may also be mediated by other effectors affecting these receptors' signaling.

### *Kummerowia striata* (Thunb.) Schindl.

*Kummerowia striata* (Thunb.) Schindl. (Fabaceae) is a flowering plant generally named Japanese clover. The ethanolic extracts have anti-inflammatory effects as tested in LPS-treated RAW264.7 cells. In these cells, *K striata* downregulated the expression of interleukin (IL)-1β, IL-6, NO, Tumour Necrosis Factor alpha (TNF-α), cyclooxygenase 2 (COX-2), upregulated IL-10 and the antioxidant factor heme oxygenase-1 (HO-1), and inhibited the production of NFκB when compared to LPS treated cells. This evidence suggests that the anti-inflammatory effects of *K. striata*’s ethanol extract were due to the downregulation of IL-1β, IL-6, nitric oxide, TNF-α, and COX-2 via the suppression of NFκB activation and upregulation of IL-10 and HO-1. The methanolic extracts of whole plant extracts of *K. striata* also showed high EA with EC_50_ values of 7.7 μg/ml (Yoo et al., 2005).

### *Lespedeza bicolor* Turcz

*Lespedeza bicolor* Turcz (Fabaceae) is a flowering plant known as shrubby bush clover, bicolor lespedeza, and shrub lespedeza. *L. bicolor* is broadly used in Australia, North America, and Eastern Asia to reduce inflammation, nephritis, hyperpigmentation, and diuresis (Ko et al., 2019). Catechin, genistein daidzein, luteolin, naringenin, and rutin were identified in the powdered extract of *L. bicolor*. The root extracts contain polyphenolic compounds that delay prostate cancer progression (Dyshlovoy et al., 2020). Also, the effects of *L. bicolor* on cognitive function were investigated in a mice model of AD using a cerebral injection of Aβ. In this study, analyzed in the following section, they identified Catechin, rutin, daidzein, luteolin, naringenin, and genistein in the powdered extract of *L. bicolor*. The methanolic extract of the stem of *L. bicolor* showed high EA with EC_50_ values of 8.6 μg/ml (Yoo et al., 2005).

### *Salvia lavandulaefolia* Vahl**.**

*Salvia lavandulaefolia* Vahl. (Laminaceae), also known as Spanish Sage, is originally from Spain and its extracts have anti-oxidant, estrogenic, anti-inflammatory properties, and anti-butyryl and anti-acetyl-cholinesterase activities (Kennedy and Scholey, 2006). The essential oil and some of its monoterpenoid components have acetylcholinesterase inhibitor (AChEI) activity. Antioxidant activity was found in the ethanolic extract of the herb (5 mg/ml) and the monoterpenoids (0.1 M) alpha- and beta-pinene and 1,8-cineole. These ethanolic extracts (50 mg/ml) and alpha-pinene and geraniol (0.2 mM), showed anti-inflammatory effects *in vitro*. The essential oil of Spanish sage (0.01 mg/ml) and its monoterpenoid component geraniol (0.1–2 mM) showed a potential EA as evidenced by the induction of β-galactosidase activity in yeast cells carrying a promoter stimulated by estrogenic compounds (Perry et al., 2001). These properties can be useful in AD, a condition characterized by a deficiency of the cholinergic system, oxidative stress, and inflammation in the brain.

### *Elettaria cardamomum* (L.) Maton

*Elettaria cardamomum* (L.) Maton (Zingiberaceae), also known as Cardamom, is used to treat menstrual disorders in India (Bhatia et al., 2015). A study tested the EA of *E. cardamomum*, *Plantago ovata* seeds, and *Gingko biloba* (GBL) and found that GBL and *E. cardamomum* seed extracts showed both EA and anti-EA (Real et al., 2015). Interestingly, another study showed that perinatal exposure to *E. cardamomum* extracts positively affected learning and memory in mice (Abu-Taweel, 2018), suggesting that it may also be helpful in AD.

### *Glycyrrhiza uralensis* Fisch.

*Glycyrrhiza uralensis* Fisch. (F. Fabaceae) is also known as Chinese Liquorice, has potent EA. Screening of plant extracts showed that *G. uralensis* induced a 300% increase in EA over basal levels (Kang et al., 2006). In addition, Liquorice is used in the Gegen Qinlian Decoction (GQD), a TCM prescribed to treat diabetes mellitus. GQD is composed of *Pueraria lobata* (Willd.) Ohwi (gen), *Scutellaria baicalensis Georgi* (huang qin), *Coptidis chinensis Franch* (huang lian), and *Glycyrrhiza uralensis Fisch* (gan cao). GQD markedly decreased blood glucose and increased serum insulin levels in type II diabetic mice (Xu et al., 2020). Since diabetes is a risk factor for AD (Jash et al., 2020), this plant's extracts could be useful to diminish the risk of AD in persons having a propensity to develop diabetes due to genetic background (Jash et al., 2020).

### *Lycopus cavaleriei* H.Lév.

*Lycopus cavaleriei* H.Lév. (syn. *Lycopus ramosissimus* (Makino) Makino) (Lamiaceae) is an estrogenic plant that in Japan is known as ko-shiro-ne, a term that means tiny white roots. Dichloromethane extraction of *L. cavaleriei* yield extracts showing EA with a RP to E2 of 0.8 × 10^–4^ (RP of E2 was 1.0) (Kim et al., 2008). The methanolic extracts of other plant of the same family, *Lycopus lucidus* Turcz. ex Benth. showed moderate EA (Kang et al., 2006).

In a recent study, the inhibitory effect of three phenylpropanoids isolated from *L. lycidus*, schizotenuin A, and lycopic acids A and B-on Aβ fibrillization was investigated using thioflavin-T assay and transmission electron microscopy (Sun et al., 2020). These phenylpropanoids, containing three cathecol groups, inhibited Aβ aggregation into amyloid plaques and the authors suggested that the catechol moieties may be crucial in reducing Aβ plaque formation (Sun et al., 2020).

A further study investigated the therapeutic potential of the prenylflavonoid xanthohumol obtained from Humulus lupulus L for AD in neuroblastoma N2a cells stably expressing human Swedish mutant amyloid precursor protein (N2a/APP), considered a cellular model of AD and HEK293/Tau cells, showing tau hyperphosphorylation. The protein analysis revealed that xanthohumol inhibited APP amyloidogenic processing to Aβ the peptide acumulation, and decreased tau hyperphosphorylation in the HEK293/Tau cells as well as in the N2a/APP by promoting protein phosphatase 2 A PP2A, and inhibiting GSK3β cells. Proteomic analysis by mass spectrometry revealed 30 proteins differentially expressed in the N2a/APP when compared to wild-type (WT) N2a cells (N2a/WT), and a total of 21 differentially expressed proteins in lysates of N2a/APP cells treated with xanthohumol. This treatment reduced AD-like changes in the proteome of N2a/APP cells, affecting proteins involved in endoplasmic reticulum stress, cytoskeleton, oxidative stress, and proteasome therapeutic potential of xanthohumol for treatment of AD and/or other tauopathies (Huang et al., 2018). Colupulone, a prenylated phloroglucinol obtained from Humulus lupulus, activates the pregnane-X-receptor (PXR), a nuclear receptor controlling the expression P-gp a protein that controls the transport troghout the Blood-brain barrier (BBB). Various colupulone analogs have been synthesized, and their effect on Aβ uptake and transport has been evaluated. Among all tested compounds, diprenylated acyl phloroglucinol displayed a significant increase (29%) in Aβ transport across bEnd3 cells grown on inserts as a BBB model. The results suggest the potential of Colupulone to enhance the clearance of Aβ across the BBB (Bharate et al., 2015).

### *Glycyrrhiza glabra* L.

*Glycyrrhiza glabra* L. (Fabaceae), also named licorice, is a well-known estrogenic plant (Zayed et al., 1964; Simons et al., 2011). An evaluation of the EA in extracts of licorice showed only weak ER and PR binding and estrogen-induced transcription of pS2 (presenilin-2), an estrogen-inducible gene present in S30 breast cancer cells (Liu et al., 2001).

Other studies investigated the effect of Glabridin a compound isolated from the roots of *Glycyrrhiza glabra* on cognitive functions and cholinesterase activity in mice. Mice were treated daily with Glabridin (1, 2 and 4 mg/kg.) for 3 consecutive days. The higher doses of glabridin significantly reduced the amnesia induced by scopolamine (0.5 mg/kg) as tested in the passive avoidance test. Furthermore, glabridin remarkably reduced the brain cholinesterase activity in mice compared to the control group. Based on the evidence they postulated that glabridin could be a promising candidate for treating memory impairment in AD patients (Cui et al., 2008).

### *Trifolium pratense* L.

*Trifolium pratense* L. (Fabaceae) known as red clover contain several polycyclic phenolic compounds, including biochanin A, genistein, daidzein, formononetin, and glycitein. Biochanin A, daidzein, genistein, and formononetin bound to the PR and AR in the range of 0.39–110 mM (Beck et al., 2003). Compounds isolated from methanolic extracts of red clover bound to the ERα and ERβ and increased alkaline phosphatase activity and the expression of PR in endometrial cells (Boue et al., 2003). Also, red clover extracts upregulated estrogen-inducible gene presenelin-2 (pS2) in S30 cells. Isolation of estrogenic components in fractionated extracts using liquid chromatography, mass spectrometry, and an EA screening bioassay showed that the isoflavone genistein was the most active phytoestrogen in the red clover extracts (Liu et al., 2001).

Preclinical studies have shown that components isolated from this estrogenic plant can be helpful in treating neuroinflammation and cognitive disorders induced by a high-fat diet in mice (Fu et al., 2019) and Aβ-induced cognitive deficits in rats (Wei et al., 2015). In this latter study, they tested the cognitive effects of an isoflavone isolated from *T. pratense* named pretensein. Cognitive impairment was induced in the experimental rats via bilateral injection of Aβ peptide into their hippocampi. The results showed that treatment with pratensein significantly decreased Aβ-induced cognitive impairment and hippocampal neurodegeneration observed in the vehicle-treated rats. Pratensein show anti-inflammatory effects significantly decreasing inflammatory biomarkers such as MDA, nitric oxide (NO), neuronal nitric oxide synthase (nNOS), IL-1β and TNF-α. Pratensein also reduced Ab levels by decreasing the expression of amyloid precursor protein (APP), and the Aβ-synthetizing enzyme β-secretase (BACE1), cathepsin B (CatB), neprilysin 2 (NEP-2), and the insulin-degrading enzyme (IDE). Moreover, pratensein significantly increased the expressions of synapse plasticity-related proteins, cAMP-response element binding protein (CREB), the brain derived neurotrophic factor (BDNF), Ca^2+^/calmodulin kinase II (CaMKII), N-methyl-D-aspartate receptor 1 (NMDAR1), postsynaptic density protein 95 (PSD-95), protein kinase A beta subunit (PKACβ), and protein kinase C gamma (PKCγ). Furthermore, pratensein significantly decreased the activity of acetylcholinesterase. The authors concluded that pratensein represents an anti-AD active compound that enhances synaptic plasticity and increases cholinesterase activity in the brain (Wei et al., 2015).

### *Sophora flavescens* Aiton

*Sophora flavescens* Aiton (Fabaceae) is commonly known as Ku shen. Methanolic root extracts of *S. flavescens* induced a 400% increase of EA in a yeast ER activity assay (Kang et al., 2006). Similarly, methanolic extracts of whole plants showed high EA (EC_50_ values of 3.2 μg/ml) (Yoo et al., 2005). In another study, the ability of both Prenylflavonoids and lavandulyl flavonoids obtained from the roots of *S. flavescens*, to displace 17 beta-estradiol (E2) from rat uterine ER was investigated. The flavonoids investigated included: 8-prenylkaempferol, leachianone A, maackiain, norkurarinone, kushenol C, kushenol X. The results showed low binding affinities of prenylated flavonoids; However, lavandulyl or prenyl groups at position 8 enhanced their binding to rat uterine ER (Hillerns and Wink, 2005). Anti-AD activities have been characterized in compounds isolated from *S. flavescens*, such as sophoflavescenol, a prenylated flavonol (Jung et al., 2011). Prenylated flavonols from *S. flavescens* inhibit the generation of advanced glycation endproducts (AGE), as well as BACE1 and the ACh degrading cholinesterases (ChE). Isolation of the active principles from *S. flavescens* by an activity-guided isolation protocol permitted to identify compound sophoflavescenol possessing high inhibitory activities against AGE, BACE1, and ChEs.

Besides, another study reported noncompetitive BACE1 inhibitory effects of lavandulyl flavanones from *S. flavescens*. Coherent with a BACE1 inhibition, these flavanones decreased Aβ release by human embryonic kidney (HEK-293) cells (Hwang et al., 2008).

### *Panax ginseng* C.A. Meyer and *Panax quinquefolius* L.

*Panax ginseng* C.A. Meyer and *Panax quinquefolius* L. (Araliaceae) known as Asian ginseng and North American ginseng, respectively. Intriguingly, extracts of *P. ginseng* and *P. quinquefolius* increased the transcription of pS2 mRNA in S30 cells; however, no significant binding to the ER or induction of PR expression was detected in the endometrial adenocarcinoma Ishikawa cells that express both types of receptors (Liu et al., 2001).

Many recent studies have shown its antioxidant and anti-inflammatory effects of *Ginseng* in the brain affected by aging-associated diseases and toxic insults such as sodium fluoride and aluminum chloride (AlCl_3_) (Mahaboob Basha and Saumya, 2013; Adam et al., 2019; Choi et al., 2021)*.* One of these studies investigated potencial pro-cognitive and neuroprotective effects of compound K, a metabolite of ginsenosides, present in the enzymatically hydrolyzed red ginseng extracts (HRGE). Amnesic mice were treated with scopolamine and tested in the passive avoidance Morris water maze and Y-maze tests. After euthanasia, the mouse’s brains were dissected and analyzed for the expression of antioxidant proteins analyzed by Western blot. The results showed that oral HRGE (300 mg/kg body weight) reversed learning and memory deficits in the scopolamine-treated mice. In the hippocampus, HRGE upregulated the nuclear-factor-E2-related factor 2 and its downstream antioxidant enzymes NAD(P)H: quinone oxidoreductase and heme oxygenase-1(HO). Treatment with HRGE also decreased glutamate excitotoxicity and ROS levels in HT22 mouse hippocampal neuronal cells. The authors proposed that HRGE alleviates cognitive impairment by preventing neuronal apoptosis triggered by oxidative stress via the upregulation of antioxidant factors such as HO (Ju et al., 2021).

Coherent with its antioxidant effects, *P . ginseng* has proven to be neuroprotective effects against Aβ toxicity in cellular and animal models of AD-like neurodegeneration in rats treated with a combination of D-Galactose and AlCl_3_. The results showed that GP (10–100 μg/ml) increased neuronal survival the mRNA expression of apoptotic proteins such as caspase-3 and Bax/Bcl-2. Also, *P. ginseng* decreased Aβ and p-tau, iNOS, MDA, and NO, and increased the cAMP levels, and the expression of SOD, phospho-PKA and phospho-CREB. The authors concluded that the neuroprotective effects of *P . ginseng* were due to the activation of the cAMP/PKA/CREB pathway.

A preparation containing ginseng known as Jangwonhwan, a boiled extract of 7 medicinal plants/mushrooms, including Korean red ginseng, has been investigated. In a preclinical study, 4.5 month-old transgenic (Tg)-APPswe/PS1dE9 AD mice (Seo et al., 2010a; Seo et al., 2010b). In one of these studies Tg mice were treated with a revised formula of Jangwonhwan 300 mg/kg/day for 3 months. LMK02-Jangwonhwan notably reduced Aβ1-42/1-40 levels, and plaque deposition, in the brain of Tg-APPswe/PS1dE9 mice. LMK02-Jangwonhwan moderately diminished oxidative stress and prevented the down-regulation of phospho-CREB and calbindin in the hippocampus. Also, when added to SH-SY5Y neuroblastoma cells, Jangwonhwan inhibited oxidative stress and Aβ-induced neurotoxicity (Seo et al., 2010b).

### *Rheum palmatum* L.

*Rheum palmatum* L. (Polygonaceae), is a plant native to southern Siberia and North and Central China. Commonly known as rhubarb, and is used as traditional medicine (Staglich, 1948). Ethanolic extracts of *R. palmatum* showed an RP of 3.85 × 10^–4^ (Zhang et al., 2005). The screening of different fractions of methanolic extracts for EA using the recombinant yeast system, consisting of a hER expression plasmid and a reporter plasmid, showed high EA levels (EC_50_ = 0.093 mg/ml) in the dichloromethane fraction. An activity-guided analysis of this fraction revealed five anthraquinones: chrysophanol, physcion, emodin, aloe-emodin, and rhein. Emodin showed the highest estrogenic RP (6.3 × 10^–2^) to 17 b estradiol RP = 1.0), and strong cytotoxicity for ER (+) MCF-7 and ER (-) MDA-MB-231 breast cancer cells (Kang et al., 2008). Rhubarb also has protective effects against Aβ toxicity in IMR-32 cells (Misiti et al., 2006).

## Estrogenic Compounds Effects on Parkinson's Disease

Parkinson's disease (PD) is a neurodegenerative disease and the second cause of dementia in the elderly. The physical examination reveals resting tremor, bradykinesia/akinesia, cogwheel rigidity, and gate impairment. The neuropathological hallmarks of PD are the appearance of intracellular aggregated forms of α-synuclein and the loss of dopaminergic neurons in the midbrain (Iarkov et al., 2020). Because there are no effective long-term treatments against PD, new therapeutic avenues are intensely searched (Danielyan et al., 2011; Danielyan et al., 2014; Jurado-Coronel et al., 2016; Mazo et al., 2017).

Some clues about potential new therapeutic avenues could arise from the epidemiological studies showing a clear difference in the PD incidence among sexes, with men being more affected than women (Jurado-Coronel et al., 2018). Estrogenic hormones such as estradiol seem to exert some protective effects against this condition; unfortunately, in addition to its beneficial effects in the brain, it may promote tumor growth in the ovary and breast. For this reason, new ER modulators, or estrogen analogs with lower activity on reproductive tissues, are being actively investigated as preventative and curative drugs for PD (Hidalgo-Lanussa et al., 2018).

### *Vicia faba* L.

*Vicia faba* L. (Fabaceae) also known as broad bean is a plant of the genus *Vicia* which members are considered to have many health benefits against PD that are attributed to several biological activities, including estrogenic, anticholinesterase, antidepressant, antioxidant, anti-inflammatory and antinociceptive, antidiabetic, anticoagulant, and antihypoxic activities (Rabey et al., 1992; Apaydin et al., 2000; Raguthu et al., 2009; Ramirez-Moreno et al., 2015; Abdel-Sattar et al., 2021; Salehi et al., 2021).

Clinical studies: After discovering the deficiency o dopamine in the striatum of patients with PD by Ehringer and Hornykiewicz in 1960, and the motor improvement with levodopa by Cotzias et al. in patients with PD, levodopa became the gold standard for treating PD. Nevertheless, this treatment resulted in motor fluctuations and dyskinesias in most patients. Many years later, a clinical study showed that Broad bean (*Vicia faba*)-a natural source of L-dopa-prolonged the "on" periods in patients with PD who have "on-off" fluctuations and shortened the “off (Apaydin et al., 2000). The study reported eight patients that benefited from broad bean meals. The patients were administered high doses of levodopa up to 800–1,000 mg per day, benefits increasing “on” time without the appearance of dyskinesias. One of the patients (Patient 1) showed a persistent response, even when they ingested broad beans only every other day. Astoundingly, in Patient 3, broad bean treatment was accompanied by a decrease of dyskinesias and a reduction of carbidopa/levodopa (L-DOPA) therapy. The authors considered that broad bean’s effects are because the patients ingested their broad bean meals garnished with yogurt rich in proteins. The authors proposed that because amino acids from dietary proteins compete with L-DOPA in crossing the BBB, they could reduce the motor effects of L-DOPA. Unfortunately, this was an unblinded trial, and a placebo effect cannot be discarded. A previous study by Rabek et al. described the acute responses following a single administration of broad beans to six patients with PD. They reported an increase in plasma L-DOPA levels after broad bean administration. This increase mirrored the motor improvements achieved by single doses of carbidopa/levodopa (Rabey et al., 1992). A similar rise of L-DOPA in plasma induced by broad bean administration was described by Vered et al. that reported that 40 g of freshly chopped broad bean provided 120–130 mg of L-DOPA (Vered et al., 1997).

### *Mucuna pruriens *(L.) DC.

*Mucuna pruriens *(L.) DC. (Fabaceae) or velvet beans (Atmagupta, Sanskrit) contain L-DOPA and have been used to treat PD in India for many years (Vaidya et al., 1978; Manyam, 1990). Several animal studies in rodents (Dhanasekaran et al., 2008) and monkeys (Lieu et al., 2012) have shown the beneficial effects of *M. pruriens* against PD-like pathology. More importantly, the therapeutic advantage compared to synthetic L-DOPA is the reduced risk of developing dyskinesias (Lieu et al., 2010).

One of the studies used a hemiparkinsonian rat model of PD to compare the effects of chronic parenteral administration of a water extract of *M. pruriens* seed powder (MPE) alone, with MPE combined with the peripheral dopa-decarboxylase inhibitor benserazide (BZ), L-DOPA alone and L-DOPA + BZ. The results were encouraging and chronic treatment with MPE alone showed significant anti-parkinsonian effects without causing dyskinesias (Lieu et al., 2010).

In a recent multicenter open trial in India, *M. pruriens*, formulated as HP-200 (Manyam et al., 2004), was administered orally to 60 patients with PD for 3 months. It was extracted as a powder from the plant seeds and administered mixed with water. The authors found that consumption of dry seeds of broad beans did not experience any clinical benefit. However, they found a significant improvement of motor abilities in the patients treated with HP-200, and hypothesized that this extract might contain L-DOPA and other antiparkinsonian compounds (Manyam et al., 2004).

## Estrogenic Plants With Antioxidant and Anti-inflammatory Activity

Estrogenic plants contain estrogenic compounds that, according to many studies including from our research groups, have antioxidant and anti-inflammatory activities that can be useful in many conditions that characterize for inflammation and oxidative stress, including neurodegenerative diseases as well as other neurological conditions (Lanussa et al., 2016; Hidalgo-Lanussa et al., 2018; Baez-Jurado et al., 2019; González-Giraldo et al., 2019; Martin-Jimenez et al., 2019; Vesga-Jimenez et al., 2019; Hidalgo-Lanussa et al., 2020).

### *Zingiber officinale* Roscoe

*Zingiber officinale* Roscoe (Zingiberaceae), commonly known as ginger, is used as a food, spice, herbal supplement for its antioxidant, anti-inflammatory and pharmacological activities (Kiyama, 2020). Epidemiological studies indicated that Ginger has beneficial effects on diabetes, metabolic syndrome, hypercholesterolemia, and inflammatory disorders (Kiyama, 2020). Ginger components include monoterpenes (cineole, citral, limonene, and alpha/beta-pinenes), sesquiterpenes (beta-elemene, farnesene, and zerumbone), phenolics compounds (gingerols, [6]-shogaol, [6]-paradol and zingerone), and diarylheptanoids (curcumin). These compounds prevented cell apoptosis, DNA damage, and inflammation by regulating cell signaling, controlling autophagy and cellular metabolism. Ginger-derived phytoestrogens are important bioactive active components modulating essential brain functions. In a previous study, ginger showed EA (RP of 3.7 × 10^–1^) (Kim et al., 2008). A methanolic extract of the plant effectively induced EA to more than 50% (Kang et al., 2006).

### *Achillea millefolium* L.

An analysis of a raw extract of the aerial parts of *Achillea millefolium* L. (Asteraceae) has EA when tested in MCF-7 cells transiently expressing ERs. After fractionating the extract, they found EA in a methanol/water fraction. From the phytoestrogens present in this extract, apigenin and luteolin activated ERs, but luteolin activated the ERβ but not the ERα (Innocenti et al., 2007). Female rats treated with acetaminophen and aqueous extracts of *A. millefollium* (2 g/kg/day) showed increased plasma levels of the toxicity marker lactate dehydrogenase. However, the extracts enhanced the activity of the antioxidant proteins reduced GSH and glutathione S-transferase (GST) in the uterus (Baggio et al., 2016). Furthermore, treatment with *A. millefollium* extracts inhibited autoimmune encephalomyelitis in mice (Vazirinejad et al., 2014).

A clinical study showed that *A. millefolium* extracts (250–500 mg/day), when administered to 75 patients with multiple sclerosis (MS) for a year, significantly delayed the time to first relapse, reduced the number of lesions, and improved their cognitive abilities (Ayoobi et al., 2019).

### *Reynoutria japonica* Houtt.

*Reynoutria japonica* Houtt. (Polygonaceae), known as Huzhang in China, is an herbaceous perennial plant natural to Eastern Asia, China, and Russia. However, several species also find in Europe and North America (Peng et al., 2013). *Reynoutria japonica* Houtt. (Polygonaceae) has been used in clinical practice for the treatment of cancer, endotoxic shock, favus, hyperlipemia, infection, inflammation, jaundice, and scald in China and Japan (Peng et al., 2013). More than 67 compounds, including quinones, stilbenes, flavonoids, coumarins, and ligands have been isolated from this plant. Ethanolic extract of *R. japonica* showed an estrogenic RP (3.28 × 10^–3^) (Zhang et al., 2005).

### *Turnera diffusa Willd. Ex Schult* **.**

This plant, known as damiana, oreganillo, or Mexican tea, grows in Bolivia, Brazil, Mexico, and North America. The leaves of *T. diffusa* contain the monoterpenes 1,8-cineole, *p*-cymene, alpha and beta-pinene, tetrafilin B, and caffeine. In the branches, also it has the flavonoid gonzalistosin, steroid beta-sitosterol, and the xacosanol alkanes *n*-triacontane and tricosan-2-one. The extracts of *T. diffusa* show antibacterial and antioxidant properties (Soriano-Melgar Lde et al., 2014). Methanolic extract demonstrated a dose-dependent inhibitory effect on aromatase activity (IC_50_ = 63.1 mg/ml). From 24 compounds tested, pinocembrin and acacetin were the most potent aromatase inhibitors having IC_50_ values of 10.8 and 18.7 mM, respectively. In addition, three compounds from the extracts apigenin 7-glucoside, Z-echinacin, and pinocembrin showed EA (Zhao et al., 2008). Previous studies have shown that *T. diffusa* has components such as arbutin that inhibit lipid peroxidation and has immunomodulatory and anti-oxidant properties that have proven beneficial for stomach ulcers (Taha et al., 2012). Anti-inflammatory activity has been found for leaf extracts of *Turnera subulata* found in the Northeastern regions of Brazil (Souza et al., 2016).

### *Cullen corylifolium* (L.) Medik.

In the TCM, *Cullen corylifolium* (L.) Medik. (Fabaceae) is also known as Buguzhi, and is used to reduce inflammatory processes. The study of ethanolic extracts of *C. corylifolium*, revealed a estrogenic RP of (1.90 × 10^–4^) (Zhang et al., 2005). The ethanolic extracts of *C. corylifolium* contained bakuchiol, psoralen, isobavachalcone, isobavachromene, and bavachinin. Also, hexane and chloroform extracts showed EA when tested in a yeast estrogen screen assay. In this assay, ethanol, hexane, chloroform extracts of CC showed EA, and bakuchiol showed similar EA than genistein (10^–6^ M). In an ER binding assay, bakuchiol displayed the strongest ER-binding affinity and five times higher affinity for ERα than for ERβ (Lim et al., 2011).

Psoralen is the main bioactive compound in the fruits of *C. corylifolium.* It is used to treat inflammation, bacterial infections (destroying the formation of biofilm), viral infections, and conditions associated with menopause such as osteoporosis (Ren et al., 2020). Psoralen’s anti-osteoporotic effect involve different proteins factors that participate in the bone remodeling such as bone morphogenetic proteins (BMPs), matrix metalloproteinases, peroxisome proliferators-activated receptor-gamma (PPARgamma), as well as signaling cascades including the inositol-requiring enzyme 1 (IRE1)/apoptosis signaling kinase 1 (ASK1)/c-jun N-terminal kinase (JNK), wnt/β-catenin, and the Protein kinase B (Akt)/activator protein-1 (AP-1) pathways (Ren et al., 2020). These signaling factors may help to treat osteoporosis by activating the osteoblast/osteoclast/chondrocyte differentiation process. The anti-inflammatory and neuroprotective effects of psoralen seem to be the result of its interaction with viral polymerase (Pol), and regulating the activation of TNF-α, transforming growth factor β (TGF-β), interleukin (IL) -4/5/6/8/12/13, GATA-3, and AChE (Ren et al., 2020).

### *Curcuma aromatica* Salisb.

*Curcuma aromatica* Salisb. (Zingiberaceae) also known as wild turmeric. An early study found EA in rhizome extracts of this plant with RP of 8.5 × 10^–4^ (Kang et al., 2006). Another study investigated the putative estrogenic and/or antiestrogenic activities of several Asiatic medicinal plants. They found that the highest EA (relative to 17 β -estradiol, RP = 1) were in this order: flowers extracts of *Pueraria lobata* (RP, 7.75 × 10^–3^), *Amomum xanthioid*es (1.25 × 10^–3^), *Glycyrrhiza uralensis*, *Zingiber officinale, Rheum undulatum, Curcuma aromatica, Eriobotrya japonica, Sophora flavescens, Anemarrhena asphodeloides, Polygonum multiflorum,* and *Pueraria lobata* roots (ranging from 9.5 × 10^–4^ to 1.0 × 10^–4^). *Prunus persica, Lycoppus lucidus,* and *Adenophora stricta* had lower RP (9.0 × 1 0^–5^ to 8.0 × 10^–5^). *Cinnamomum cassia* and *Prunus persica*, showed antiestrogenic effects with RP of 1.14 × 10^–3^ and 7.4 × 10^–4^, respectively (tamoxifen RP = 1) (Kim et al., 2008).

In addition, Curcuminoids from* C. aromatica* have neuroprotective activities against oxidative stress. Curcuminoids present in the radix of *C. aromatica* are neuroprotective against the toxic effects of 24 h-treatment with H_2_O_2_ in PC12 cells. The curcumoids increased cell survival, decreased the release of LDH (a measure of cell death), the cellular Ca^2+^, and ROS levels, enhanced the activities of antioxidant proteins such as SOD, catalase (CAT) and GSH, stabilized the mitochondrial membrane potential, and improved PC12 morphology. The chemical analysis showed that diarylheptanoids and sesquiterpenoids are the primary active chemicals in the extracts tested. On the other hand, Curcumin is the main component of *Curcuma longa*, a Chinese medicinal plant used to alleviate brain disorders, which is neuroprotective and increases the survival of cultured rodent cortical neurons.

The protective effects of curcumin were blocked when neurons were pretreated with a tyrosine kinase B (TrkB) antibody, known to inhibit the activity of the brain-derived neurotrophic factor (BDNF). Conversely, treatment with curcumin increased BDNF and phospho-TrkB effects that, in turn, were blocked by ERK and PI3K inhibitors. Curcumin activated ERK, CREB, and AKT, by mechanisms involving MAPK and PI3K. The authors concluded that curcumin beneficial effects might be mediated via the BDNF/TrkB-MAPK/PI-3K-CREB pathway (Wang et al., 2010).

### *Eriobotrya japonica* (Thunb.) Lindl

*Eriobotrya japonica* (Thunb.) Lindl. (Rosaceae) commonly known as the Japanese Medlar or loquat. A study reported that methanolic extracts of *E. japonica* induced an increase of EA (>50%) (Kang et al., 2006). Ethyl acetate fractions of *E. japonica* showed EA with an RP of 6.1 × 10^–2^ (RP E2 = 1.0) (Kim et al., 2008). However, it my be used with caution as seeds, or pips, and young leaves are slightly poisonous because contain little amounts of cyanogenic glycosides such as amygdalin which release cyanide if eaten. However, numerous studies have shown powerful anti-inflammatory effects in different disease conditions. Silver nanoparticles of *Eriobotrya japonica* AgNPs stimulated phagocytosis and prevent allergies and the authors concluded that *E. japonica* could be a promising therapy for preventing inflammation, and bacterial infection by enhancing of phagocytosis (Jabir et al., 2021), arthritis (Kuraoka-Oliveira et al., 2020), asthma (Kim T.-M. et al., 2020), intestinal problems, hepatic damage induced by acetaminophen (Jiang et al., 2017), and metabolic syndrome induced by a high fat diet in C57BL/6J mice (Li F. et al., 2020). In the most recent study male mice 7–9 weeks of age were randomly divided into three groups (*n* = 6 mice/group). The first treatment group received 250 µl of saline by IP injection, the second received 50 µg LPS via IP and group 3 was pre-treated with AgNPs (1 mg/kg) and then received 50 µg LPS. After 10 h, mice were sacrificed, and blood and peritoneal fluid was taken for analysis of IL-1β and IL-6 levels. The results showed that the AgNPs synthesized using E. japonica leaf extract inhibited the LPS-induced synthesis and the levels of cytokines such as IL-1β and IL-6 (Jabir et al., 2021).

### *Vigna radiata* (L.) R.Wilczek

*Vigna radiata* (L.) R.Wilczek (Fabaceae) is known as mung bean sprout, common in the tropics and warm temperature zones and its extracts have shown to increase cell proliferation above the levels induced by estradiol. This proliferation was suppressed by the ER antagonist, ICI 182.780, suggesting that ER signaling pathways are mediating the proliferative effects of *V. radiata* (Boue et al., 2003).

Preclinical studies have investigated anti-inflammatory and antiarthritic activity of *V. radiata* sprouts in rats. Ethanolic extracts of the sprouts were used to evaluate its antiarthritic activity using rats treated with complete Freund's adjuvant as a model of arthritis. Treatment with ethanolic extracts of *V. radiata* significantly reduced the biochemical changes induced by the Freund's adjuvant, such as lipid peroxidation, total reduced glutathione, myeloperoxidase and lysosomal enzymes like cathepsin-D, N-acetyl beta-D-glucosaminase and beta-D-glucuronidase. The findings of this study suggest that *V. radiata* can reduce inflammation in arthritis (Venkateshwarlu et al., 2016). Further studies are required to investigate its effects in other inflammatory conditions.

### *Wurfbainia villosa* var. *Xanthioides* (Wall. ex Baker) Skornick. and A.D.Poulsen

*Wurfbainia villosa* var. *Xanthioides* (Wall. ex Baker) Skornick. and A.D.Poulsen, commonly known as bastard cardamom and Malabar Cardamom, is a medicinal plant native to Indonesia that is used to treat spleen and stomach diseases in China (An et al., 2020). Butanolic fractions of *W. villosa* var. *xanthioides* showed EA with a relative potency of 6.6 × 10^–2^ (RP of E2 = 1.0) (Kim et al., 2008). Also, methanolic extract of this plant effectively induced a 50% increase in EA (Kang et al., 2006).

The herbal mix named CG (plus) contains *Artemisia gmelinii* Weber ex Stechm. (syn, *Artemisia iwayomogi* Kitamura), *W. villosa* and *Salvia miltiorrhiza Bunge*. The effect of CG (plus) on stress for immobilization (IS) was recently assessed in mice. Male BALB/c mice were daily treated with different doses of aqueous extracts of CG (plus) (0–200 mg/kg) for 5 days. On the last day, mice were subjected to IS for 6 h. Stress increased corticosterone levels, adrenaline, cytokines, and the enzyme markers of hepatic injury (ALT and AST) in serum. The mix CG (plus) significantly prevented the effects of stress (Kim H.-G. et al., 2020).

### *Iris domestica* (L.) Goldblatt & Mabb.

*Iris domestica* (L.) Goldblatt & Mabb. syn *Belamcanda chinensis* (Iridaceae) commonly known as Blackberry Lilly. The ethanolic extract of *I. domestica* showed EA with a RP of (1.26 × 10^–4^) (Zhang et al., 2005). Isoflavonoids obtained from a methanolic extract of *I. domestica* rhizomes inhibited mutagenesis when tested in the Ames test and have antioxidant effects. The isoflavonoids reduced transition-metal ions and prevented the peroxidation of polyunsaturated fatty acids. The isoflavones, identified included the glycosides tectoridin and iridin and the aglycones irigenin, tectorigenin, and 5,6,7,3′-tetrahydroxy-4′-methoxyisoflavone (Wozniak et al., 2010).

### *Senna obtusifolia* (L.) H.S.Irwin and Barneby

*Senna obtusifolia* (L.) H.S.Irwin and Barneby (syn. *Cassia obtusifolia* L.) (Fabaceae) is commonly known as American sicklepod. *S. obtusifolia* is a weed that grows in the world's tropical and subtropical regions, even when the environmental conditions are unfavorable. This plant has been investigated as a laxative and sexual stimulant in male rats (Ajayi et al., 2014). Ethanolic extracts of *S. obtusifolia* have shown estrogenic RP of 3.49 × 10^–4^ (Zhang et al., 2005). However, its toxic effects observed in cattle and mice discouraged its use as a medicinal plant (Furlan et al., 2014; Ana Maria et al., 2019).

A different study investigated the effects of Aurantio-obtusin, an anthraquinone isolated from dried seeds of *S. obtusifolia* and *Cassia* tora L. (syn. *Senna* tora). Aurantio-obtusin was tested as an anti-inflammatory compound on lipopolysaccharide- (LPS)-induced inflammation in RAW264.7 cells. ELISA and quantitative real-time PCR (qRT-PCR) assays revealed that this compound significantly decreased the production of NO and PGE_2_ by inhibiting the expression of COX-2 and iNOS, as well as the cytokines TNF-α, and IL-6. Aurantio-obtusin showed anti-inflammatory effects *via* the inhibition of NFκB in RAW264.7 cells. These results suggested that this compound could have therapeutic value against inflammatory diseases.

### *Medicago sativa* L.

*Medicago sativa* L. (Fabaceae) is commonly known as alfalfa sprout. *M. sativa* extracts induce more cell proliferative effects than estradiol. The estrogen receptor antagonist ICI 182,780 (Fulvestrant) suppressed the cell proliferation induced by the extracts, suggesting that ERs were involved (Boue et al., 2003). Moreover, the phytoestrogens present in these extracts exhibited selectivity toward ERβ (Boue et al., 2003). Also a recent study showed that Alfalfa extracts have dose-dependent beneficial effect on nicotine-induced oxidative damage and neuroinflammation in the brain as well as reducing anxiety (Raeeszadeh et al., 2021). In this study the authors investigated the effect of the hydroalcoholic extract of *M. sativa* on brain damage and anxiety behavior in male Wistar rats exposed to nicotine. Rats were randomly divided into six treatment (T) groups: T1 and T2 were subcutaneously injected with 250 and 500 mg/kg alfalfa extract, respectively, T3 and T4 groups where animals were injected subcutaneously with 0.2 mg/kg nicotine and 250 and 500 mg/kg alfalfa extract, and T5 group was injected with 0.2 mg/kg nicotine. After treatments, the serum levels of the cytokines TCA, IL-1, and TNFα and oxidative stress-defensive factors such as gluthation (GPx, SOD, total antioxidant capacity (TAC), and MDA) in the brain were assayed. The time and number of entrances in the open arm significantly decreased in the treated groups in a dose-depended manner. Oxidative stress parameters were higher in the nicotine -treated rats and lower in the rats treated with 500 mg/kg alfalfa extracts (T2). Nicotine increased the levels of the cytokines TNFα and IL-1 in the T5 group when compared to the other groups. More importantly, treatment with the alfalfa extracts increased the levels of the antioxidant factors GPx and SOD and decreased the number of necrotic neurons and gliosis in the nicotine-treated rats (Raeeszadeh et al., 2021).

### *Phaseolus vulgaris* L.

*Phaseolus vulgaris* L.(Fabaceae) is also known as green bean or red kidney bean (Boue et al., 2011; de Lima et al., 2014). *P. vulgaris* produces phytoalexins (isoflavones) under stress conditions such as wounding and/or insect damage. Thus to induce the expression of flavonoids, the components of methanolic extracts of red kidney bean that were treated with the fungus *Aspergillus sojae,* and untreated controls plants were tested for estrogenic activity using an ERE luciferase reporter assay (Boue et al., 2011). The estrogenic activities of the phytoalexins kievitone and phaseollin were isolated from fungus-treated red kidney bean extracts and analyzed for EA using ERα and ERβ binding and ERE luciferase and cell proliferation assays in MCF-7 and HEK 293 cells. These phytoalexins stimulated MCF-7 cell proliferation and Kievitone showed a higher binding affinity to ERα and Phaseollin to ERβ. Although phaseollin displayed attenuation of ER transactivation in the ERE luciferase assay in MCF-7 cells, both phytoalexins attenuated the effects of E2 in an MCF-7 cell survival assay showing estrogenic and antiestrogenic activities (Boue et al., 2011). The green bean extracts were more effective in inducing cell proliferation than estradiol and exhibited a preferential affinity for ERβ. The proliferative effects were due to its estrogenic effects as it was suppressed by ICI 182,780 (Boue et al., 2011). *P. vulgaris* has shown antioxidant activity in both cell and animal studies (Venkateswaran et al., 2002; Venkateswaran and Pari, 2002; Ampofo and Ngadi, 2020). One recent study, investigated phenolic compounds, and antioxidant activity, of different species of the genus Phaseolus. The species closest to each other in terms of essential amino acid content were *P. polyanthus* with *P. vulgaris* and *P. lunatus* with *P. coccineus*. Previous studies show a positive correlation between antioxidant activity and flavonoids, anthocyanins, and lectins. Significant differences in the content of phenolic compounds have been found among the bean species. No obstant, in addition to *P. vulgaris*, other species such as *P. coccineus* and *P. lunatus* have high antioxidant potential (Alcazar-Valle et al., 2020).

### *Pueraria montana* var. *lobata* (Willd.) Maesen and S.M.Almeida ex Sanjappa and Predeep

*Pueraria montana* var. *lobata* (Willd.) Maesen and S.M.Almeida ex Sanjappa and Predeep (Fabaceae), also known as kudzu, is a perennial leguminous vine used as an herbal medicine in Asia, containing flavonoids that are concentrated in its root (Eom et al., 2018). Methanolic extract of flowers of *P. lobata* increased the EA > 50%, but roots only showed moderate EA (Kang et al., 2006). Extracts of kudzu roots induced cell proliferation by a mechanism involving ER as it was suppressed by the antagonist, ICI 182,780 (Boue et al., 2003). Ethanolic extracts of *Pueraria lobata* showed an estrogenic RP of (6.17 × 10^–5^) (Zhang et al., 2005)**.** In another study, 17β-estradiol showed EA with an EC_50_ = 0.205 ± 0.025 ng/ml (RP = 100) and methanolic extracts of flowers and stem of *P. montana* var. *lobata* showed EA with EC_50_ values of 8.6–1.0 μg/ml, respectively (Yoo et al., 2005).

A study that used peritoneal macrophages isolated from BALB/c mice and cells activation with lipopolysaccharide (LPS) or LPS plus interferon (IFN)-gamma revealed that kudzu leaf and its principal active compound robinin (kaempferol-3-O-robinoside-7-O-rhanmoside) have anti-inflammatory activity. The extract inhibited the macrophage synthesis of the inflammatory factors iNOS, COX-2, TNFα, and IL-6. Kudzu extracts decreased LPS-induced activation of (JNK and TANK-binding kinase 1 (TBK1). Also, kudzu extract inhibited LPS/IFN-gamma-induced expression of the signal transducer and activator of transcription 1 (STAT1). Robinin decreased iNOS protein involving modulation of JNK and STAT1 activation. But without any impact on other inflammatory markers such as NFκB (Eom et al., 2018).

## Other Estrogenic Plants

Many other plants with demonstrated EA require further characterization include: *Adenophora stricta* Miq. (Campanulaceae), native to East Asia (China), also known as Sha Shen and Ladybells (Kim et al., 2008); *Artemisia vulgaris L.* (Asteraceae), often named mugwort, contains the flavonoids eriodictyol and apigenin (Lee et al., 1998); *Asplenium trichomanes* L. (Aspleniaceae) known as maidenhair spleenwort, it is used to control irregular menses and as an emmenagogue, aborticide, expectorant, anti-cough remedy, and laxative (Dall'Acqua et al., 2009); *Hylodesmum podocarpum* subsp. *oxyphyllum* (DC) H. Ohashi and R.R.Mill. syn. *Desmodium oxyphyllum* (Fabaceae). This plant, also called ticktrefoils, is a medicinal plant from Asia, that is used for breast cancer (Yoo et al., 2005); *Maackia amurensis*-Rupr (Fabaceae), is a tree also known as Amur maackia (Yoo et al., 2005; Grishchenko et al., 2016); *Humulus lupulus *L. (Cannabaceae) Methanolic plant extracts bind to ERα and ERβ. And it has EA. Numerous studies of the estrogenic properties for hops are attributed to the component 8-prenylnarigenin (Liu et al., 2001). Another study compared the estrogenic effects of hops to other plants of the licorice species (*Glycyrrhiza glabra*, *G. inflata*, and *G. uralensis*) (Hajirahimkhan et al., 2013). All extracts showed EA when tested in cultured endometrial cancer cells, MCF-7 cells expressing an ERE directing the expression of the fluorescent reporter luciferase. The authors ordered the plant extracts by decremental EA as follows: *H. lupulus* > *G. uralensis* > *G. inflata* > *G. glabra*. Liquiritigenin was selective for ERβ, and isoliquiritigenin was equipotent for both ERα/β. 8-Prenylnaringenin showed nanomolar estrogenic RP without ER selectivity (Hajirahimkhan et al., 2013). A recent study identified 8-Prenylapigenin from *Glycyrrhiza inflata* as an ERβ agonist (Hajirahimkhan et al., 2018).

## Conclusions and Future Perspective

The deficit of estrogen is involved in the development or progression of various types of autoimmune diseases, cancer (breast, colorectal, endometrium, ovary, prostate), cardiovascular diseases, osteoporosis, obesity, insulin resistance, and neurodegenerative diseases and mood disorders. The deficit in sex hormones such as estrogen negatively impacts the cognitive abilities, general health, and consequently the quality of life of millions of persons around the world. HRT positively impacts cognitive abilities and immune function, but its benefits seem to be restricted to the early stages of menopause. After menopause, HRT with estrogens combined with progestins has shown beneficial effects on climacteric symptoms and osteoporosis in the bone and on body weight. However, it increases the risk of mammary carcinomas and cardiovascular diseases. Consequently, plant-derived alternatives are currently investigated. The advancement of our understanding of the molecular mechanisms underlying the effects of plant-derived estrogenic compounds will facilitate the discovery of new therapies to counteract the adverse effects of a deficit of estrogen in the brain. Interestingly, many plants used in traditional medicine have shown estrogenic activity that is concurrent with antioxidant, antiproliferative, and anti-inflammatory activities.

The discovery of estrogenic compounds from medicinal plants with lower side effects should permit to improve the cognitive abilities in women affected by menopause and other conditions inducing oxidative stress and neurodegeneration without the side effects elicited by actual HRT.

Recently encouraging clinical results with a combination of herbs have shown to be effective and safe in decreasing menopause symptoms (Rattanatantikul et al., 2020). Also, phytoestrogens and their metabolites from medicinal plants have been shown to mimic the benefits of endogenous estrogens, and estrogen-based therapies are considered promising approaches for preventing the onset and progression of neurodegenerative disorders (Bustamante-Barrientos et al., 2021). Though, xenoestrogens derived from plastic polycarbonates, such as water bottles and varnish coating in food cans, can alter estrogen-mediated signaling pathways inducing a negative effect on health. It may be required to counteract the estrogen deficit in a more holistic approach comprising an adequate diet containing natural estrogenic compounds, body and mental exercise practices that promote neurogenesis, a more ecologically friendly way of life, and adequate social and spiritual support systems to decrease the effects of stress. On the other hand, the explosive development of new genetic therapies may permit the modulation of specific estrogenic receptors in the brain, bones, and other tissues without increasing the risk of disease while preventing the pathological effects of lower sex hormones' levels.

## Glossary

AAB: Anemarrhena asphodeloides Bunge
Aβ: Amyloid beta petide
AChEI: acetylcholinesterase inhibitors
AD: Alzheimer's disease
AGE: advanced glycation endproducts
APOE: Apolipoprotein E
APP: Aβ precursor protein
AR: androgen receptor
BDNF: brain-derived neurotrophic factor
BMD: bone mineral density
BMP: bone morphogenetic protein
CAT: catalase
ChE: cholinesterases
COX-2: cyclooxygenase 2
CRE: cAMP response element
DBT: Danggui Buxue Tang
ER: estrogen receptor
ERE: estrogen-responsive element
ERK: extracellular-regulated kinase
GBL: Gingko biloba
GFAP: glial fibrillar acidic protein
GQD: Gegen Qinlian Decoction
GSH: reduced glutathione
GST: glutathione S-transferase activity
hER: human ER
HRT: hormone replacement therapy
IL: Interleukin
l-DOPA: levodopa
LH: Luteinizing hormone
MENQOL: menopause-specific QOL
MMSE: Mini-Mental State Examination
MS: multiple sclerosi
NFκB: nuclear factor kappa B
NO: nitric oxide
NOS: nitric oxide synthase
OVX: ovariectomized
PKA: protein kinase A
PKB: protein kinase B
PKC: protein kinase C
PS1: presenilin 1
PR: progesterone receptor
QOL: quality of life
RP: relative potency
SHBG: sex hormone binding globulin
Tg: transgenic
TNF-α: tumor necrosis factor alpha
YCJ: young coconut juice
