# Supplementary material for: Estrogenic Plants: to Prevent Neurodegeneration and Memory Loss and Other Symptoms in Women After Menopause
Source: Front Pharmacol. 2021 May 20;12:644103. doi: 10.3389/fphar.2021.644103 (PMC8172769; doi:10.3389/fphar.2021.644103)
Supplement: Supplementary file 1 [file Table1.DOCX]

| Table.1. Traditional medicinal Plants with Estrogenic activity | | | | | | | |
| --- | --- | --- | --- | --- | --- | --- | --- |
|  | **Scientific name** | **Family** | **Common name** | **Extract/ compound** | **Model** | **Effect** | **Reference** |
| 1 | *Achillea millefolium L.* | *Asteraceae* | Yarrow | Methanolic  Extract | MCF-7 cells | Stimulation of the Estrogen Receptor (ER) β | (Innocenti et al., 2007) |
| 2 | *Adenophora stricta Miq.* | *Campanulaceae* | Sha Shen and Ladybells | Methanolic extract | Recombinant yeast system | EA | (Kim et al., 2008) |
| 3 | *Anemarrhena asphodeloides Bunge* | *Asparagaceae* | Zhi mu | Methanolic extract | Recombinant yeast system | EA | (Kim et al., 2008) |
| 4 | *Angelica sinensis (Oliv.) Diels* | *Apiaceae* | Dong quai, dang gui | Ferulic Acid and Z-Ligustilide | Ovariectomized (OVX) Rat | EA | (Liu et al., 2001) (Circosta et al., 2006) |
| 5 | *Artemisia vulgaris L.* | *Asteraceae* | Mugwort | Eriodictyol and apigenin | *Saccharomyces cerevisiae* strain BJ3505 | EA | (Lee, 1998) |
| 6 | *Asplenium trichomanes L.* | *Aspleniaceae* | Maidenhair spleenwort, Tie jiao jue | Leaves infusion, decoction and methanol extract | MCF7/EREluc cell line which expresses endogenous ERα, and SK-NBE cells transiently transfected with the Erα and ERβ | Stimulation of ERα and ERβ | (Dall'Acqua et al., 2009) |
| 7 | *Astragalus mongholicus Bunge. syn. Astragalus membranaceus* | *Fabaceae* | Mongolian Milk, Huang qi | Decoction | MCF-7 cells | Activation of estrogen-responsive element (ERE) | (Gong et al., 2016) |
| 8 | *Elettaria cardamomum L.* | *Zingiberaceae* | Cardamon | Methanolic extract | MCF-7 breast cancer cells using the E-Screen bioassay | EA | (Real et al., 2015) |
| 9 | *Cocos nucifera L.* | *Arecaceae* | Coconut palm | Juice | Ovariectomized rat | EA on the progression of AD-like pathology | (Radenahmad et al., 2009) |
| 10 | *Cullen corylifolium (L.) Medik* | *Fabaceae* | Bu gu zhi, Cule | Ethanolic extract | *Saccharomyces cerevisiae* ER + LYS 8127, MCF-7 cells | Stimulation of Erβ y Erα | (Lim et al., 2011) |
| 11 | *Cynomorium coccineum subsp. songaricum (Rupr.) J. Léonard* | *Cynomoriaceae* | Hongo de Malta and Jopo de lobo | Ethanolic extract | Recombinant yeast system | EA | (Zhang et al., 2005) |
| 12 | *Curcuma aromatica Salisb.* | *Zingiberaceae* | Yu jin, wild turmeric | Rhizome extract | Recombinant yeast system | EA | (Kang et al., 2006) |
| 13 | *Dunbaria villosa (Thunb.) Makino* | *Fabaceae* | Ye bian dou | Methanolic extract | Ishikawa cells (endometrial cells) | EA | (Yoo et al., 2005) |
| 14 | *Dunbaria villosa (Thunb.) Makino* | *Fabaceae* | Ye bian dou | Methanolic extract | Ishikawa cells | EA | (Yoo et al., 2005) |
| 15 | *Epimedium brevicornu Maxim.* | *Berberidaceae* | Yin yang huo, Horney goat weed | Ethanolic extract | Recombinant yeast system | EA | (Zhang et al., 2005) |
| 16 | *Glycine max (L.) Merr.* | *Fabaceae* | Soybean, Da dou, Soya | Methanolic extract/ Biochanin A, genistein, daidzein, formononetin, and glycitein | MCF-7 cells | EA, binding to the ERα and ERβ and the progesterone receptor (PR) | (Boué et al., 2003) |
| 17 | *Glycyrrhiza uralensis Fisch.* | *Fabaceae* | Gan cao, Chinese liquorice | Ethanolic extract | Recombinant yeast system | EA | (Kang et al., 2006) |
| 18 | *Glycyrrhiza glabra L.* | *Fabaceae* | Licorice, Yang gan cao, Regaliz | Ethyl acetate extract from licorice root | Recombinant yeast system | EA towards either one or both ERα and ERβ). | (Simons et al., 2011) |
| 19 | *Humulus lupulus L.* | *Cannabaceae* | Hops, Pi jiu hua | Methanolic extract | Ishikawa cells | Competitive binding to ERα and ERβ. | (Liu et al., 2001) |
| 20 | *Hylodesmum podocarpum subsp. oxyphyllum*  *(DC.) H.Ohashi & R.R.Mill. syn. Desmodium oxyphyllum* | *Fabaceae* | Jian ye chang bing shan ma huang, Tick-trefoils | Methanolic extract | Ishikawa cells | EA | (Yoo et al., 2005) |
| 21 | *Iris domestica*  *(L.) Goldblatt & Mabb. syn. Belamcanda chinensis* | *Iridaceae* | Vanilla domestica, blackberry lilly | Ethanolic extract | Recombinant yeast system | EA | (Zhang et al., 2005) |
| 22 | *Kummerowia striata (Thunb.) Schindl.* | *Fabaceae* | Ji yan cao, Japanese clover | Methanolic extract | Ishikawa cells | EA | (Yoo et al., 2005) |
| 23 | *Lespedeza bicolor Turcz.* | *Fabaceae* | Shrub bush-clover, bicolored lespedeza, shrub lespedeza, Hu zhi zi | Methanolic extract | Ishikawa cells | EA | (Yoo et al., 2005) |
| 24 | *Lycopus cavaleriei H. Lév. syn. Lycopus ramosissimus* | *Lamiaceae* | Xiao ye di su, Ko-shiro-ne | Dichloromethane extraction | Recombinant yeast system | EA | (Kim et al., 2008) (Kang et al., 2006) |
| 25 | *Maackia amurensis Rupr.* | *Fabaceae* | Amur maackia, Chao xian huai | Methanolic extract | Ishikawa cells | EA | (Yoo et al., 2005) |
| 26 | *Maackia floribunda (Miq.) Takeda syn. Maackia fauriei* | *Fabaceae* | Hanemi-inuenju, Duo hua ma an shu | Methanolic extract | Ishikawa cells | EA | (Yoo et al., 2005) |
| 27 | *Medicago sativa L.* | *Fabaceae* | Alfalfa sprout, Zi mu xu | Methanolic extract/ Biochanin A, genistein, daidzein, formononetin, and glycitein | MCF-7 cells | EA- Binding to the ERα and ERβ and the progesterone receptor (PR) | (Boué et al., 2003) |
| 28 | *Panax ginseng C.A. Mey. and Panax quinquefolius L.* | *Araliaceae* | Asian ginseng and North American ginseng, respectively. | Methanolic extract | Ishikawa cells | Competitive binding to estrogen receptors alpha (ER alpha) and beta (ER beta). | (Liu et al., 2001) |
| 29 | *Phaseolus vulgaris L.* | *Fabaceae* | Green bean Red kidney bean | Methanolic extract | MCF-7 cells | Estrogenic and antiestrogenic activities. | (Boué et al., 2011) |
| 30 | *Prunus persica (L.) Batsch* | *Rosaceae* | Peach, melocotón | Methanolic extract | Recombinant yeast system | EA | (Kang et al., 2006) |
| 31 | *Pueraria montana var. lobata (Willd.)* | *Fabaceae* | Ge ma mu, Kudzu root | Methanolic extract | Recombinant yeast system Cultured MCF-7 cells Ishikawa cells | EA-Binding to the ERα and ERβ and PR | (Kang et al., 2006, Boué et al.2003,Yoo et al.,2005) |
| 32 | *Reynoutria japonica Houtt. syn. Fallopia japonica and Polygonum cuspidatum.* | *Polygonaceae* | Hu zhang | Ethanolic extract | Recombinant yeast system | EA | (Zhang et al., 2005) |
| 33 | *Reynoutria multiflora(Thunb) Moldenke syn Polygonum multiflorumThunb).* | *Polygonaceae* | Chinese knotweed, He shou wu, Fo-ti | Ethanolic extract | Recombinant yeast system | EA | (Zhang et al., 2005) (Kang et al., 2006) |
| 34 | *Rheum palmatum L.* | *Polygonaceae* | Chinese Rhubard, Zhang ye da huang | Ethanolic extract | Recombinant yeast system | EA | (Zhang et al., 2005) (Kang et al., 2008) |
| 35 | *Rheum rhabarbarum L.* | *Polygonaceae* | Wine-plant, Rhubarb | Ethanolic extract | Recombinant yeast system | EA | (Kang et al., 2006) (Kim et al., 2008) |
| 36 | *Rhodiola rosea L.* | *Crassulaceae* | Golden root, Rose root, Arctic root, Orpin rosea, Hong jing tian | Extract (flavonoids) | Molecular docking | EA (ERα y Erβ) | (Powers and Setzer, 2015) |
| 37 | *Salvia officinalis L.* | *Lamiaceae* | Sage, Garden sage, Salvia | Aqueous-ethanolic extract | Cell line T47D-Kbluc | Estrogenicity (ERLUX assay) | (Rahte et al., 2013) |
| 38 | *Salvia lavandulifolia Vahl* | *Lamiaceae* | Spanish Sage | Ethanolic extract | Recombinant yeast system | EA | Perry et al., 2001) |
| 39 | *Scutellaria baicalensis Georgi* | *Lamiaceae* | Huang qin, Chinese skullcap | Ethanolic extract | Recombinant yeast system | EA | (Zhang et al., 2005) |
| 40 | *Senna obtusifolia (L.) H.S. Irwin & Barneby. syn Cassia obtusifolia L.* | *Fabaceae* | American sicklepod | Ethanolic extract | Recombinant yeast system | EA | (Zhang et al., 2005) |
| 41 | *Sophora flavescens Aiton* | *Fabaceae* | Ku shen | Ethanolic extract | Recombinant yeast system & Ishikawa cells | EA | (Kang et al., 2006) (Yoo et al., 2005) |
| 42 | *Trifolium pratense L.* | *Fabaceae* | Red clover, Hong che zhou cao | Methanolic extract/ Biochanin A, genistein, daidzein, formononetin, and glycitein | MCF-7 cells | EA Binding to the ERα and ERβ and the progesterone receptor (PR) | (Boué et al., 2003) |
| 43 | *Turnera diffusa Willd.* | *Passifloraceae* | Damiana, Oreganillo, Mexican Tea | Methanolic extract | Yeast estrogen screen (YES) assay. | EA | (Zhao et al., 2008) |
| 44 | *Vitex agnus-castus L.* | *Lamiaceae* | Vitex, chaste tree, chasteberry | Methanolic extract | Ishikawa cells | Competitive binding to estrogen receptors alpha (ER alpha) and beta (ER beta). | (Liu et al., 2001) (Liu et al., 2004) |
| 45 | *Vigna radiata (L.)* | *Fabaceae* | Lu dou, Mung bean | Methanolic extrac Biochanin A, genistein, daidzein, formononetin, and glycitein | MCF-7 cells | EA Binding to the ERα and ERβ and the progesterone receptor (PR) | (Boué et al., 2003) |
| 46 | *Wurfbainia villosa var. xanthioides, syn. Amomum xanthioides*  *(Wall ex Baker) Skornick. & A.D. Poulsen (syn, Amomum xanthioides Wallich)* | *Zingiberaceae* | Malabar cardamom | Methanolic extract | Recombinant yeast system | EA | (Kim et al., 2008) |
| 47 | *Zingiber officinale Roscoe* | *Zingiberaceae* | Jiang, Ginger | Methanolic extract | Recombinant yeast system | EA | (Kim et al., 2008) (Kang et al., 2006) |
