# Supplementary material for: Estrogenic Plants: to Prevent Neurodegeneration and Memory Loss and Other Symptoms in Women After Menopause
Source: Front Pharmacol. 2021 May 20;12:644103. doi: 10.3389/fphar.2021.644103 (PMC8172769; doi:10.3389/fphar.2021.644103)
Supplement: Supplementary file 2 [file Table2.DOCX]

| **Table 2.** **Cognitive and neuroprotective effects of phytoestrogens** | | | | |
| --- | --- | --- | --- | --- |
| **Common name** | **Extract/compound** | **Assay** | **outcome** | **Reference** |
| Soy | Isoflavones | *In vivo* (rats) | Soy phytoestrogens functioned as estrogenic agonists regulating the RNA coding for the ChAT and NGF in the brain of female rats. | (Pan et al., 1999) |
|  | Isoflavones (genistein, daidzein, and glycitein) | *In vivo* (humans) | ↑ Executive function; language | (File et al., 2001) |
|  | Soy isoflavones | *In vivo* (humans) | Enhanced memory and the function of the frontal lobe in postmenopausal women | (File et al., 2001) |
|  |  |  | Enhanced memory, attention, working memory, and language | (File et al., 2005) |
|  | Phytoestrogens |  | Potential neuroprotective effects by attenuating neuroinflammation | (Jantaratnotai et al., 2013) |
|  | Isoflavones (genistein, daidzein, and glycitein) | *In vivo* (human) | Improved psychomotor speed, memory, executive functions, and language abilities | (Kritz-Silverstein et al., 2003) |
|  | Isoflavones | *In vivo* (human) | Enhanced executive function, attention, working memory, and language abilities | (Duffy et al., 2003) |
|  |  | *In vivo (human)* | Improved working memory performance in a dose-dependent manner | (Pan et al., 2000) |
|  |  | *In vivo* (rats) | Improved spatial memory and increased spine density | (Luine et al., 2006) |
|  |  | *In vivo* (rats) | Enhanced visual and spatial memory in adult rats | (Lund et al., 2001) |
|  |  | *In vivo* (human) | Improved short-term memory in adult rats | (Lee et al., 2004) |
|  |  | *In vivo* (human) | Increased psychomotor speed, attention, working memory, and visuospatial reasoning | (Casini et al., 2006) |
|  |  | *In vivo* (human) | Increased speed processing in menopausal women | (Greendale et al., 2012) |
|  | Isoflavones (genistein, daidzein, and glycitein) | *In vivo* (human) | Ameliorated attention, working memory, and visuospatial memory | (Fournier et al., 2007) |
|  | Isoflavones (genistein and daidzein) | *In vivo* (human) | Improved psychomotor speed, memory, attention, working memory, and visuospatial abilities | (Gleason et al., 2009) |
|  | Isoflavones (equol) | *In vivo* (human) | No global cognitive benefits were observed; however, positive correlations between, Equol level and speeded dexterity and verbal fluency were found in Patients with AD. | (Gleason et al., 2015) |
|  | Isoflavones (daidzein and genistein, and glycitein) | *In vivo* (human) | Increased visual memory in postmenopausal women | (Henderson et al., 2012) |
|  |  | *In vivo* (human) | No cognitive benefits, but they enhanced the quality of life in all subscales (vasomotor, psychosexual, physical, and sexual) in postmenopausal women | (Basaria et al., 2009) |
|  | Isoflavones 80 mg | *In vivo* (human) | No effects on cognition or quality of life of Chinese postmenopausal women | (Ho et al., 2007) |
|  | Isoflavones | *In vivo* (human) | No positive effects in men with prostate cancer and androgen deprivation therapy | (Sharma et al., 2009) |
|  | Isoflavones and lignan (polyphenolic compounds with EA) | *In vivo* (human) | A high intake of lignan was associated with superior performance in mental processing capacity and speed, as well as executive function abilities in aged Dutch women | (Kreijkamp-Kaspers et al., 2007) |
|  | Isoflavones | *In vivo* (human) | A possible beneficial effect of isoflavone on some abilities of the central executive in menopausal women (50 to 65 years of age, n=38) | (Santos-Galduroz et al., 2010) |
|  | Phytoestrogens | *In vivo* (rats) | Improved visual-spatial memory in female rats and inhibited it in male rats | (Lund et al., 2001) |
| Hibiscus sabdariffa calyces | Plant extract | *In vivo* (rats) | Improved spatial memory and hippocampal BDNF expression in the hippocampus of ovariectomized rats | (Lorenzana-Martinez et al., 2020) |
| Red clover | Isoflavones enriched fraction | *In vivo* (human) | Neuroprotective t against glutamate toxicity in human cortical neurons | (Occhiuto et al., 2008) |
| Soy/ Red clover | Biochanin A | *In vitro* and *In vivo* | Neuroprotective against lipopolysaccharide-induced neuronal damage. Inhibited microglia activation and prevented the damage of dopaminergic neurons, both in vivo and *in vitro*. Decreased the levels of interleukin (IL)-1β, IL-6, TNF-α, and inhibited the generation of ROS. | (Wang et al., 2016b) |
|  | Genistein |  | Protect dopaminergic neurons by reducing microglia activation | (Wang et al., 2005) |
|  |  |  | Perinatal exposure improved spatial learning and memory, but impaired passive avoidance learning and memory of rat offspring | (Kohara et al., 2014) |
|  |  |  | Improves spatial memory in male rats with high glucose levels | (Kohara et al., 2015) |
|  | Daidzein |  | Diminished the lipopolysaccharide (LPS)-induced release of inflammatory mediators by BV-2 microglial cells | (Occhiuto et al., 2008) |
|  |  |  | Significantly attenuated malondialdehyde (MDA) content but decreased glutathione concentration in the brain of rats | (Choi, 2006) |
|  | Resveratrol | Clinical trial | It has enhanced both cerebrovascular function and cognition in postmenopausal women. | (Evans et al., 2017) |
|  | Resveratrol (75 mg twice daily) | Clinical trial | Improved cerebrovascular function, mood, and cognition in postmenopausal women | (Evans et al., 2016) |
| Fusarium spp. | α-zearalanol | *In vivo* (mice) | Ameliorates memory and decreased DNA oxidative damage in the brain of ovariectomized mice | (Dong et al., 2013) |
|  |  | *In vivo* (mice) | Improved memory and neurogenesis in ovariectomized mice | (Dong et al., 2014) |
|  |  | *In vitro*  *(cells)* | Showed neuroprotective effect in differentiated PC12 cells by activating the estrogen receptor alpha | (Dong et al., 2015) |
| Achillea millefolium (yarrow) | Aqueous extract | *In vivo* (rats) | Restored memory and reduced anxiety behavior induced by brain ischemia via ERs in ovariectomized rats | (Jahromi, 2019) |
| Achillea fragrantissima | Phytochemicals | *In vivo Transgenic* APPswe/PS1DeltaE9 mouse model of AD | Decreased APP metabolism and slightly improved memory in the novel object recognition task | (Bartolotti et al., 2018) |
| yarrow | Apigenin | *In vitro* | Inhibits activated microglial cells and neuronal death in animal models of AD | (Elsisi et al., 2005) (Rezai-Zadeh et al., 2008) |
|  |  | *In vitro* | protect copper-mediated β-amyloid neurotoxicity | (Zhao et al., 2013b) |
|  |  | *In vitro* | Inhibited the disaggregation of alpha-synuclein oligomers | (Caruana et al., 2012) |
|  |  | *In vivo* (mice) | improves the locomotor and muscular activity and protected dopaminergic neurons | (Patil et al., 2014) |
|  | Luteolin | *In vitro* | Inhibits Aβ-induced neuronal death in mouse cortical cultures | (Choi et al., 2014) |
|  | Luteolin plus Palmitoylethanolamide | *In vitro and Ex vivo* (hippocampus, mice) | Reduced the expression of Nitric oxide synthase NOS, glial fibrillar protein GFAP, restored BDNF, and inhibited apoptosis | (Paterniti et al., 2014) |
|  | Luteolin | *In vitro* | Inhibits memory impairment, the degeneration of cholinergic neurons, and amyloid deposition in a mouse model of AD | (Wang et al., 2016a) |
|  |  |  | Decreased AD-like pathology after traumatic brain injury in Tg2576 mice | (Sawmiller et al., 2014) |
|  |  |  | Protectedneural pheochromocytoma (PC12) and glial C6 cells against MPTP+ toxicity | (Wruck et al., 2007) |
|  | Nephrocizin  (Luteolin-7-O-beta-D-glucopyranoside) | *In vitro* | Protected differentiated PC12 cells against 6-hydroxydopamine toxicity ( a cell model of PD)soy | (Lin et al., 2012) |
|  | Luteolin and apigenin | *In vivo* | Neuroprotection and neurotrophic activity in mice with MPTP-induced Parkinsonism | (Patil et al., 2014) |
|  | Kaempferol | *In vitro* | Inhibits formation, extension, and destabilization of beta-amyloid fibrils in Escherichia coli | (Sharoar et al., 2012) |
|  |  |  | Inhibited Aβ-induced toxicity in mouse-derived hippocampal neuronal cells (HT22) as well as PC12 and T47D cells | (Yang et al., 2014); (Roth et al., 1999) |
|  |  | *In vitro/In vivo (mice)* | Reversed Abeta-induced neurotoxicity in PC12 cells and cognitive impairment in ICR mice | (Kim et al., 2010) |
|  |  | *In vitro* | Prevented oxidative stress-induced cell death in rat PC12 and primary neuronal cells | (Qu et al., 2009) |
|  |  | *In vivo (mice)* | Improved motor coordination, raise striatal dopamine level, and prevent the loss of dopaminergic neurons in a mouse model of PD | (Li and Pu, 2011) |
